# Co-binding by YY1 identifies the transcriptionally active, highly conserved set of CTCF-bound regions in primate genomes

**DOI:** 10.1186/gb-2013-14-12-r148

**Published:** 2013-12-31

**Authors:** Petra C Schwalie, Michelle C Ward, Carolyn E Cain, Andre J Faure, Yoav Gilad, Duncan T Odom, Paul Flicek

**Affiliations:** 1 European Molecular Biology Laboratory, European Bioinformatics Institute, Wellcome Trust Genome Campus, Hinxton, Cambridge CB10 1SD, UK; 2 University of Cambridge, Cancer Research UK-Cambridge Institute, Robinson Way, Cambridge CB2 0RE, UK; 3 Current address: Department of Human Genetics, University of Chicago, Chicago, IL 60637, USA; 4 Wellcome Trust Sanger Institute, Wellcome Trust Genome Campus, Hinxton CB10 1SA, UK; 5 Current address: Laboratory of Systems Biology and Genetics, Institute of Bioengineering, School of Life Sciences, École Polytechnique Fédérale de Lausanne (EPFL), Lausanne CH-1015, Switzerland

## Abstract

**Background:**

The genomic binding of CTCF is highly conserved across mammals, but the mechanisms that underlie its stability are poorly understood. One transcription factor known to functionally interact with CTCF in the context of X-chromosome inactivation is the ubiquitously expressed YY1. Because combinatorial transcription factor binding can contribute to the evolutionary stabilization of regulatory regions, we tested whether YY1 and CTCF co-binding could in part account for conservation of CTCF binding.

**Results:**

Combined analysis of CTCF and YY1 binding in lymphoblastoid cell lines from seven primates, as well as in mouse and human livers, reveals extensive genome-wide co-localization specifically at evolutionarily stable CTCF-bound regions. CTCF-YY1 co-bound regions resemble regions bound by YY1 alone, as they enrich for active histone marks, RNA polymerase II and transcription factor binding. Although these highly conserved, transcriptionally active CTCF-YY1 co-bound regions are often promoter-proximal, gene-distal regions show similar molecular features.

**Conclusions:**

Our results reveal that these two ubiquitously expressed, multi-functional zinc-finger proteins collaborate in functionally active regions to stabilize one another's genome-wide binding across primate evolution.

## Background

CTCF is a highly conserved, 11-zinc finger multi-functional protein [1,2] important in regulating gene expression [3-5], insulating against enhancer-promoter interactions [6,7], regulating splicing [8], as well as ensuring allele-specific expression at imprinted genes [7] and on the inactive X chromosome [9]. Genome-wide studies have suggested that CTCF binding demarcates active and repressive domains [10-12] and contributes to nucleosome positioning [13], as well as nuclear organization and higher order chromatin structure [14].

CTCF's binding profile is largely (but not entirely [15]) invariant across mouse tissues [16], human cell lines [10] and divergent species compared to those of tissue-specific transcription factors (TFs) [17-22]. Comparisons of CTCF binding have revealed a high level of conservation in liver tissue of species separated by up to 180 million years [21], as well as in cell lines from human, mouse, and chicken [19]. Additionally, CTCF has been shown to bind transposable elements in both embryonic stem cells [18] and differentiated tissue [21]. While certain repeat elements have expanded CTCF target sites in several mammalian lineages, thus far there is no evidence of this process being prevalent in primates based on experiments in human and rhesus macaque [21].

The availability of sequenced primate genomes [23-26] and the ability to transform blood B cells into immortal lymphoblastoid cell lines (LCLs) with the Epstein-Barr virus (EBV) [27] facilitates functional genomics comparisons across different primate species. To date, such inter-primate studies have been carried out primarily at the level of gene expression [28-32]. However, it had already been proposed in the 1970s that phenotypic differences between primates are largely due to regulatory differences [33]. While comparative evolutionary studies in mammals have provided insight into regulatory mechanisms, limited information is available within the primate order.

Inter-primate comparisons of regulatory evolution have been performed for histone modifications, which can explain 7% of gene expression differences among human, chimpanzee, and rhesus macaque cell lines [34]. Further, DNA methylation studies revealed that promoter methylation differences underlie 12 to 18% of gene expression differences between humans and chimpanzees and that approximately 10% of CpG islands are significantly differentially methylated between the two species [35,36]. Differences in the binding of transcriptional regulators have been inferred from the presence of several hundred species-specific DNase I hypersensitive sites near genes differentially expressed between humans and chimpanzees [37]. Regulatory DNA element comparisons among primates are emerging [38,39]; however, a comprehensive analysis of the binding of a sequence-specific factor such as CTCF across primate species has yet to be performed.

CTCF can exert its different functions through interactions with diverse protein factors [40,41]. One such factor is Yin Yang 1 (YY1), which was originally shown to trans-activate the *Tsix* ncRNA during X-chromosome inactivation through its interaction with CTCF [9]. There is a strong pattern of co-localization between these two factors at predicted boundary elements, suggesting that they could act synergistically in delimiting chromatin domains [42]. Genome-wide chromatin immunoprecipitation followed by high-throughput sequencing (ChIP-seq) data have recently indicated global co-localization of CTCF and YY1 in human cells [43] with a specific distance constraint [44].

YY1 was first identified as both a repressor and an activator of the adeno-associated virus under different conditions [45], but, similar to CTCF, it has been attributed a broad range of distinct functions, including roles in imprinting [46-48], X-chromosome inactivation [49], and chromatin structure maintenance [50]. YY1 is essential in mouse development, as its deletion results in peri-implantation lethality [51]. A homolog of YY1, the *Drosophila* PHO protein, is involved in Polycomb repression [52,53], but there is limited evidence for this in mammals, where YY1 is rather viewed as a global regulator. YY1 binding motifs are overrepresented in core promoters [54], with approximately 10% of human promoters containing it [55]. Additionally, YY1 is important for initiating transcription of various transposable elements such as LINE-1s [56,57], Alu SINEs [58], Herv-Ks [59] and LTRs [60].

Here, we map genome-wide CTCF binding at high resolution in seven primate species and propose that the evolutionary stability of CTCF genomic occupancy is, at least in part, linked to its co-binding with the ubiquitous TF YY1.

## Results

### Evolution of CTCF binding in seven primates

**Figure 1:**
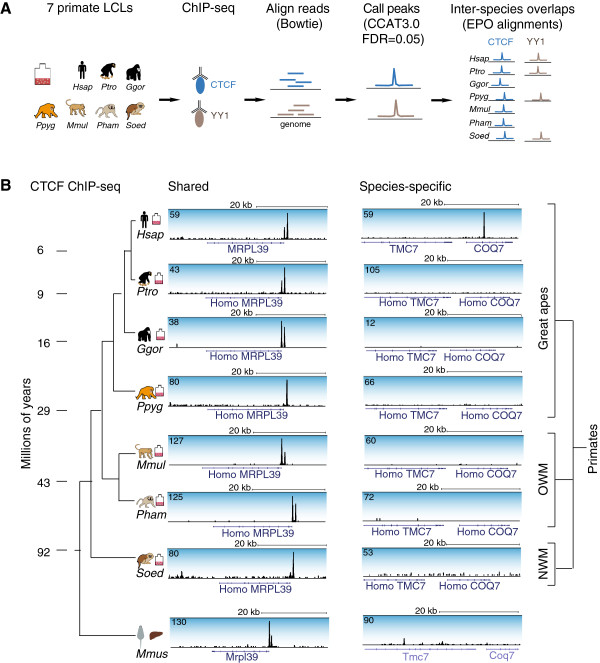
**CTCF ChIP-seq in seven primate species. (A)** Lymphoblastoid cell lines (LCLs) from seven primate species: *Homo sapiens* (*Hsap*), *Pan troglodytes* (*Ptro*), *Gorilla gorilla* (*Ggor*), *Pongo pygmaeus* (*Ppyg*), *Macaca mulatta* (*Mmul*), *Papio hamadryas* (*Pham*) and *Sanguinus Oedipus* (*Soed*) were formaldehyde cross-linked prior to CTCF ChIP-seq experiments. YY1 ChIP-seq was performed in a subset of primates - *Hsap*, *Ptro*, *Ppyg* and *Soed*. Sequencing reads were aligned to each respective species' genome and peaks called at a fixed false discovery rate (FDR). Inter-species comparisons were based on the EPO multiple sequence alignments. **(B)** A primate-shared CTCF binding event at the *MRPL39* gene, and a human-specific binding event within the *COQ7* gene in LCLs from each primate species [representing the great ape, Old World monkey (OWM) and New World monkey (NWM) clades] as well as *Mus musculus* (*Mmus*) liver.

CTCF binding in distantly related mammalian species is highly conserved compared to that of tissue-specific TFs [17-22]. Here, we analyzed the evolution of CTCF binding at high resolution in LCLs from seven primates, spanning 40 million years of evolution, to determine what mechanism(s) contribute to binding conservation. We experimentally profiled CTCF in most of the great apes (*Homo sapiens* - *H.sap*, *Pan troglodytes* - *P.tro*, *Gorilla gorilla* - *G.gor*, and *Pongo pygmaeus* - *P.pyg*), two species of Old World monkey (*Macaca mulatta* - *M.mul* and *Papio hamadryas* - *P.ham*), and one species of New World monkey (*Sanguinus oedipus* - *S.oed*) (Table S1 in Additional file 1). In each species, we performed ChIP-seq in at least two replicates and used naked DNA (input) as control (Figure 1A; Additional file 1: Table S2). Species were aligned to their respective genome except for *P.ham*, which was aligned to the *M.mul* genome (84% reads aligned), and *S.oed*, which was aligned to the *Callithrix jacchus* (*C.jac*) genome (65% reads aligned), as there are currently no published genomes available for these species, and *M.mul* and *C.jac* represent the closest sequenced relatives. We determined regions of significant ChIP enrichment (referred to as 'bound regions' or 'binding events') compared to input samples with CCAT 3.0 (Materials and methods) using a fixed false discovery rate (FDR) of 0.05 across species (Materials and methods). Inter-species comparisons were made using Ensembl release 60 genome-wide 6-way EPO primate multiple alignments [61], which include *H.sap*, *P.tro*, *G.gor*, *P.pyg*, *M.mul* and *C.jac* (Table S1 in Additional file 1). Our universal cutoff approach is unbiased in that it does not assume that binding events are conserved across species; however, it favors a model where differences are more likely than shared binding and as such is likely to marginally underestimate the fraction of conserved binding events [21,22] (Materials and methods).

**Figure 2:**
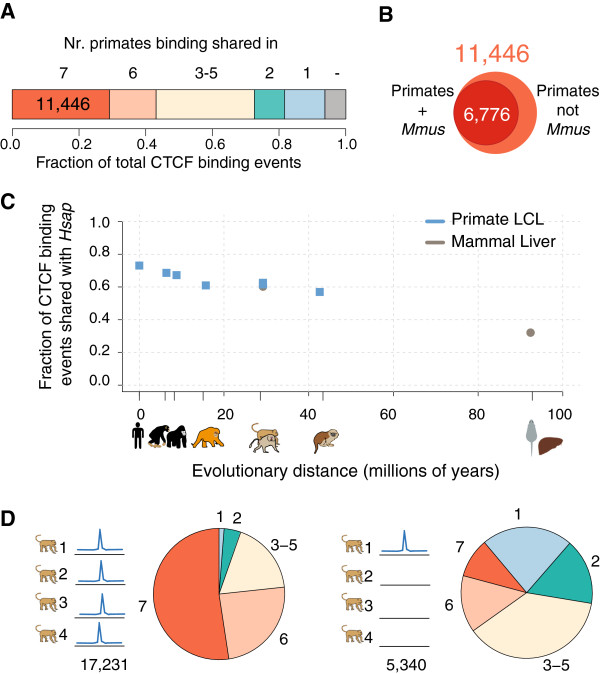
**CTCF binding is highly conserved across primates. (A)** Categorization of CTCF binding events on the basis of their evolutionary conservation. Fraction of all CTCF-bound regions across species that are not contained within the EPO multiple alignments (−), present in a single species (1) and shared among two, three to five, six, or all seven primate species. **(B)** The fraction of primate-shared CTCF binding events (LCL) found in mouse (liver). **(C)** The fraction of CTCF binding events in each species overlapping human CTCF binding events. Species are ordered by evolutionary divergence time (millions of years). *H.sap*, *P.tro*, *G.gor*, *P.pyg*, *M.mul*, *P.ham*, *S.oed* LCL CTCF binding data (blue); *H.sap M.mul* and *M.mus* liver CTCF binding data [21] (grey). The human fraction is based on inter-individual overlaps. **(D)** Regions bound in four macaque cell lines (left) or one macaque cell line (right) have different patterns of conservation between species (colored as in panel **A**).

In each of the analyzed primates, we detected thousands of shared and species-specific CTCF-bound locations (Figures 1B and 2A). To determine the extent of binding conservation, we split CTCF-bound regions into six different classes: (i) species-specific and not included in the genome-wide multiple alignments, (ii) species-specific and included in the alignments, (iii) shared with exactly one other species, (iv) shared with two, three, or four other species, (v) shared with exactly five other species (that is, missing in exactly one species), and (iv) shared across all primates (Figure 2A; Figure S2A in Additional file 1). On average, approximately 40% of CTCF-bound regions are shared across six or seven species (highly shared). Conversely, thousands of regions (on average 20% of all CTCF binding events) are species-specific or only shared with a single other species. The vast majority (>80%) of CTCF-bound regions are shared between at least two species and 11,446 binding events are shared across all analyzed primates (Figure 2A; Figure S2A,B in Additional file 1). Of the binding events common to seven primates, 98% are also bound in human liver (11,256 regions), the majority of which are also bound in mouse liver (6,776 regions; Figure 2B; Figure S2C in Additional file 1). In other words, relatively few CTCF binding events are exclusively found in primates, and not in other mammalian lineages.

The differences in CTCF binding accumulate in line with the evolutionary distance between compared species, as has been observed for more distant mammals [21], and pairwise binding overlap fractions between each primate species and human correlate negatively with evolutionary distance (Pearson's r = −0.92, *P* = 0.004; Figure 2C; Figure S2B in Additional file 1). Pairwise conservation estimates are consistent with previously published comparisons of CTCF binding in rhesus macaque and mouse liver [21] as well as human and gorilla LCLs [26]. Similarly, as expected based on prior reports [21,26], highly shared binding events show stronger ChIP enrichment, a better match to the consensus motif and a higher overlap with other cell types/tissues (in this case, liver) than species-specific binding events (Figure S2D-G in Additional file 1).

In order to determine the relationship between inter-individual and inter-species variation, we analyzed independently derived LCLs from *H.sap* (two LCLs), *P.tro* (three LCLs) and *M.mul* (four LCLs). While over three-quarters of the regions bound by CTCF in all four probed *M.mul* cell lines are also shared with five or six other primates, less than one-quarter of cell line-specific bound regions show such high overlap (Figure 2D). Conversely, binding events shared across seven species are present in the vast majority (over 80%) of the individual LCLs, in contrast to roughly half of CTCF-bound regions unique to one or two species. (Figure S2H in Additional file 1). These results indicate that regions bound by CTCF across individuals are more likely to be evolutionarily conserved than individual-specific bound regions. In sum, CTCF binding that is unstable between species also tends to be unstable between individuals within a species, and vice versa.

### Evolutionarily conserved CTCF-bound regions are co-bound by YY1

**Figure 3:**
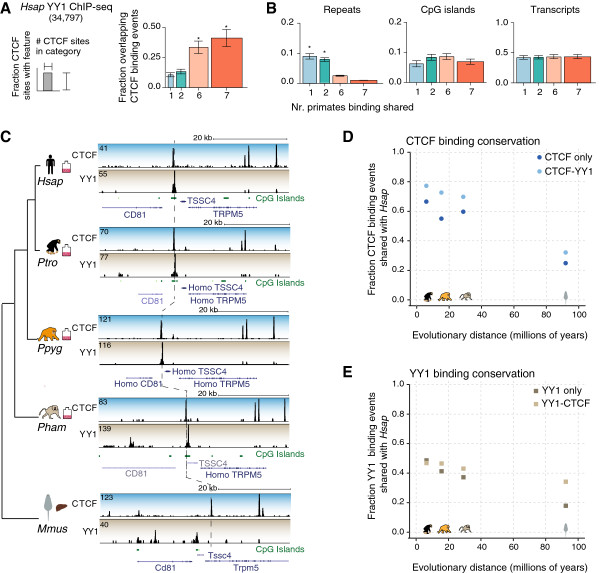
**Evolutionarily conserved CTCF-bound regions are highly associated with YY1. (A)** Fraction of CTCF binding events in each conservation category associated with YY1 binding in human LCLs [43]. Error bars represent the standard error between at least three cell lines. *P < 0.05, Wilcoxon rank-sum test. **(B)** Fraction of CTCF binding events in each conservation category associated with repetitive elements, CpG islands, and transcript annotations across all primate species. Error bars represent the standard error across seven species. *P < 0.05, Wilcoxon rank-sum test. **(C)** Conserved CTCF and YY1 binding events at the *CD81* and *TSSC4* genes in *H.sap*, *P.tro*, *P.pyg*, and *P.ham* LCLs. The CTCF binding event is also present in *M.mus* liver, while the YY1 binding event is not. **(D)** Fraction of CTCF-only (dark blue) and CTCF-YY1 (light blue) binding events in each species overlapping the corresponding regions in *H.sap*. **(E)** Fraction of YY1-only (dark brown) and YY1-CTCF (light brown) binding events in each species overlapping the corresponding regions in *H.sap*.

As combinatorial binding of TFs can stabilize regulatory regions [62], and as CTCF has previously been shown to co-localize and functionally interact with the TF YY1 [42-44,51], we asked whether co-binding with YY1 could help explain high CTCF binding conservation. Indeed, we found that almost half (41%) of the primate-shared regions are also bound by YY1 in human LCLs compared to less than 20% of species-specific CTCF binding events (Figure 3A). CTCF-YY1 co-bound regions enrich for all-primate shared CTCF binding regardless of the proximity to transcription start sites (Figure S3A in Additional file 1). In contrast, less than 15% of the regions bound by tissue-specific TFs such as NFKB and Pax5 are also bound by CTCF, irrespective of evolutionary class (Figure S3B in Additional file 1). Evolutionarily conserved CTCF-bound regions are not specifically enriched for repetitive elements, CpG islands or transcripts (Figure 3B).

In order to establish whether YY1 co-binding stabilizes CTCF binding in evolution, we performed YY1 ChIP-seq experiments in *H.sap*, *P.tro*, *P.pyg* and *P.ham* LCLs, as well as in primary liver tissue from human and mouse. YY1 binds tens of thousands of locations in all interrogated primates, as well as in mouse liver (Figure S3C in Additional file 1). Almost 10,000 regions bound by YY1 are shared across the four primates included in this analysis (4-way shared), 61% of which are also bound in human liver and 40% of which are shared with mouse liver (Figure S3C in Additional file 1). In comparison, virtually all of the 18,000 4-way shared CTCF LCL binding events are present in human liver, and approximately 50% of these are also bound in mouse liver. In other words, for both CTCF and YY1, about half of the binding events found in multiple primate species are also bound in mouse liver. Overall, pairwise YY1 binding conservation is typically lower than observed for CTCF (Figures S2B and S3D in Additional file 1). For instance, *H.sap* and *P.tro* share 48% of YY1 binding events compared to 69% of CTCF binding events, *P.tro* and *P.pyg* 62% YY1 versus 66% CTCF and *P.pyg* and *P.ham* 59% versus 71% bound regions. Nevertheless, like CTCF, YY1 binding is more highly conserved than that observed for tissue-specific TFs such as CEBPA and HNF4A [22].

After assessing CTCF and YY1 binding independently, we combined the two datasets to analyze the stability of regions co-bound by CTCF and YY1 (Figure 3C-E). CTCF binding events that co-localize with YY1 (CTCF-YY1) in one species are more likely to be shared with a second species (in this case human), and a similar effect is observed for YY1-bound regions that co-localize with CTCF (Figure 3D,E); reflecting this, we observed stronger sequence conservation of CTCF-YY1 co-bound locations (Figure S3E in Additional file 1). For each pair of species, the regions co-bound by CTCF and YY1 are consistently more evolutionarily stable than those bound by either one of the factors in isolation.

In summary, regions co-bound by CTCF and YY1 show enhanced sequence conservation and are more likely to exist and be bound in a second mammalian species, at both shorter and wider evolutionary distances.

### Binding to repeat elements by either CTCF or YY1 does not explain most species-specific regions

As both CTCF and YY1 have previously been shown to bind to and expand species-specifically via repetitive elements [18,21,56-60] we analyzed the association between binding events and the repetitive genome. We found that CTCF-YY1 bound regions are less likely to overlap annotated repeats than either CTCF-only or YY1-only regions (*P* < 0.05; Figure S3F in Additional file 1), which is not unexpected given their high conservation. In order to determine the extent to which repetitive elements contribute to species-specific binding, we further analyzed CTCF and YY1 binding events independently.

**Figure 4:**
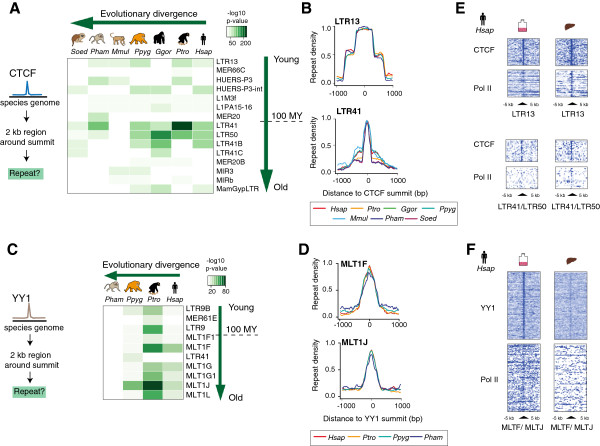
**CTCF and YY1 bind to distinct repetitive elements across primate species with no evidence of lineage-specific expansions. (A)** Significant CTCF binding event associations with repeat elements. MY, million years ago. **(B)** LTR13 and LTR41 repeat profiles centered on the summit of significantly associated CTCF binding events across primate species. **(C)** Significant YY1 binding event associations with repeat elements. Pol II, RNA polymerase II. **(D)** MLT1F and MLT1J repeat profiles centered on the summit of significantly associated YY1 binding events across primate species. **(E)** CTCF and Pol II ChIP-seq binding profiles in a 10 kb window around LTR13 and LTR41/LTR50 repeat elements in *H.sap* LCLs and *H.sap* liver. **(F)** YY1 and Pol II ChIP-seq binding profiles in a 10 kb window around MLT1F/MLT1J repeat elements in *H.sap* LCLs and *H.sap* liver. For panels A and C only associations with a -log *P*-value >10 in at least one species are shown; the color intensity represents the association's significance. Repeat elements are sorted by estimated age from youngest to oldest and primate species by evolutionary distance from human.

First, we identified the annotated repetitive elements in each species bound by CTCF (Figure 4A; Figure S4A and Table S3A in Additional file 1). We detected few species-specific repeat associations, but observed consistent enrichments of older mammalian repeats, such as LTR41 and LTR50, as well as overrepresentation of CTCF binding to primate-specific repeats, such as LTR13 [63,64] (Figure 4B). Most were members of the LTR repeat family, consistent with previous evidence of functional exaptation of LTRs in primates (Figure S4A in Additional file 1) [65].

We also searched for repeat-specific CTCF motif words, which previously revealed CTCF-bound repeat expansions in more diverse mammalian species [21]. We detected no species-specific motif words and only a limited number of words bound at a higher frequency in primates than in other mammalian species (Figure S4B in Additional file 1). LTR13 was again identified as a CTCF-bound primate-specific repeat; however, less than 300 binding events account for this enrichment across primates, a comparatively low number considering the tens of thousands of B3-specific motif words that have shaped the CTCF binding landscape in rodent genomes [21].

We similarly identified which annotated repetitive elements are associated with YY1 binding. MLT1-type repeats of the ERVL-MaLR family, including MLT1F and MLT1J, are found to be significantly associated with YY1 binding (Figure 4C,D; Figure S4D and Table S3B in Additional file 1). MLT1-type repeats are not enriched in YY1 binding events in human and mouse livers, suggesting that the genomic interaction of YY1 and this repeat class might be tissue-specific, unlike the observed LTR repeat association with CTCF binding events (Figure 4E,F). Finally, we did not find an enrichment of repeat-embedded YY1 binding at or in close proximity to repeat-associated CTCF binding locations, indicating that these factors bind distinct repeats. Thus, repeats do not appear to be involved in CTCF-YY1 co-binding in the genome.

In sum, active repeat expansions have not substantially contributed to the CTCF binding repertoire in seven major primate lineages, and most species-specific CTCF and YY1 binding events do not appear to be mediated by repeat elements.

### YY1 couples CTCF binding to transcriptional activity

**Figure 5:**
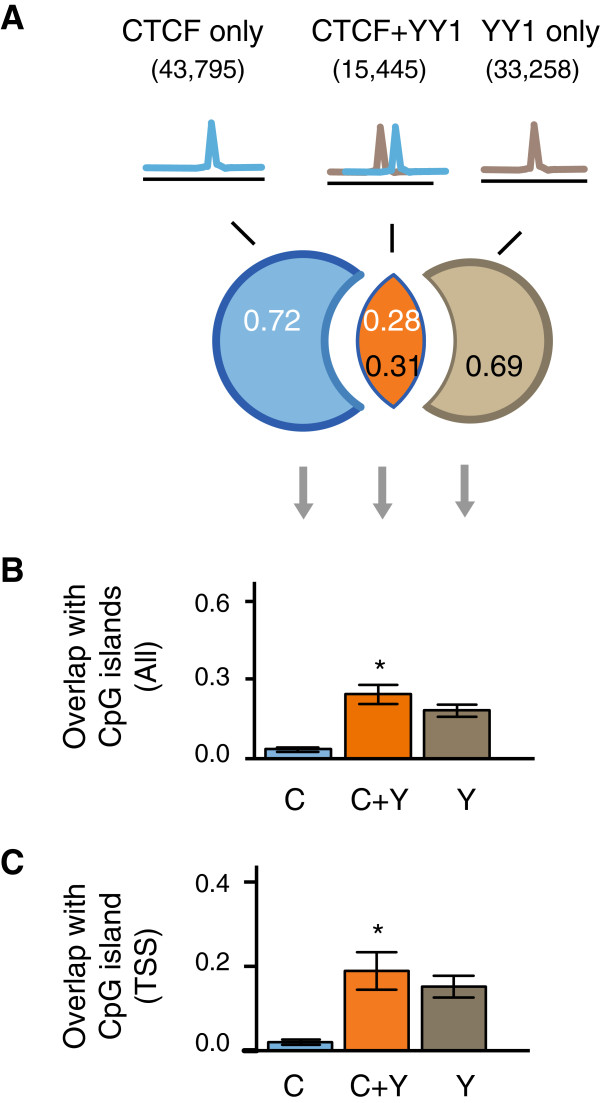
**CTCF-YY1 co-bound regions associate with CpG islands. (A)** The fraction of overlapping CTCF- and YY1-bound regions in *H.sap*, *P.tro*, *P.pyg*, and *P.ham* LCLs. **(B)** The fraction of CTCF-only, CTCF-YY1 and YY1-only binding events overlapping CpG islands across species. **(C)** The fraction of CTCF-only (C), CTCF-YY1 (C + Y) and YY1-only (Y) binding events overlapping CpG island transcription start sites (TSS) across species. Error bars represent standard error of the mean among four species. *P < 0.05, Wilcoxon rank-sum test, C versus C + Y.

In primate LCLs, on average approximately one-third of CTCF-bound regions are co-bound by YY1, and nearly half of YY1-bound regions are co-bound by CTCF (Figure 5A; Figure S5A in Additional file 1). We asked whether any molecular or sequence features (aside from repeat elements) could differentiate isolated CTCF binding events from those co-bound by YY1. Overall, the binding intensities of CTCF at regions co-bound by YY1 are no greater than those of isolated CTCF binding events, suggesting that the observed pattern is not simply driven by ChIP enrichment class (*P* > 0.05; Figure S5B in Additional file 1). *De novo* motif discovery identified the canonical motifs for both CTCF and YY1 at shared CTCF-YY1 bound locations, indicating that both factors directly bind to DNA in general (Figure S5C in Additional file 1). However, we did not observe a consistent spacing constraint between the two motifs at co-bound regions (data not shown). Importantly, we found CTCF-YY1 co-bound regions to be significantly more associated with CpG islands (*P* < 0.05) and CpG island promoters (*P* < 0.05) than isolated CTCF binding events, indicating that co-bound regions may be more transcriptionally active (Figure 5B,C; Figure S5D in Additional file 1).

**Figure 6:**
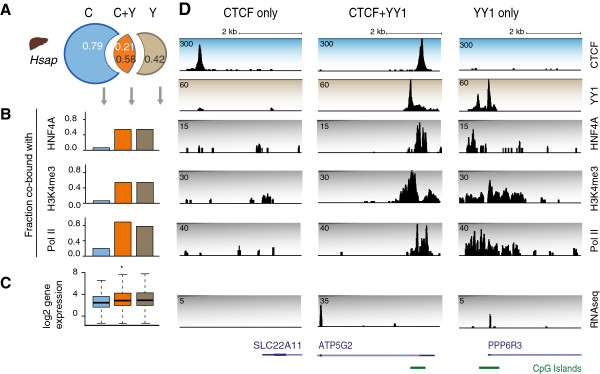
**CTCF-YY1 co-bound regions are associated with marks of active transcription. (A)** The fraction of overlapping CTCF- and YY1-bound regions in *H.sap* liver. **(B)** Association of CTCF-only, CTCF-YY1 and YY1-only binding events with a tissue-specific TF (HNF4A), Pol II, and the active H3K4me3 histone modification [21,66]. Data are representative of two individuals. **(C)** Expression level of genes overlapping CTCF-only, CTCF-YY1 and YY1-only binding events in *H.sap* liver [22]. RNA-seq data from three individuals. *P < 0.05, Wilcoxon rank-sum test, C versus C + Y. **(D)** CTCF, YY1, HNF4A, H3K4me3, Pol II and RNA-seq profiles at a CTCF-only, CTCF-YY1 and YY1-only binding event.

To further explore the relationship between transcription and conservation of CTCF-YY1 co-bound regions, we analyzed the ChIP-seq data from mouse and human liver with corresponding functional data for basal transcriptional machinery, tissue-specific TFs, and histone marks [22,66]. We found that CTCF-YY1 co-bound regions overlap marks of transcriptional activity, including RNA polymerase II (RNA Pol II), the active H3K4me3 histone modification, as well as liver-specific transcriptional regulators such as HNF4A and CEBPA (Figure 6A,B; Figure S6A,B in Additional file 1). In contrast, CTCF-bound regions lacking YY1 (CTCF-only) rarely co-localize with marks of active transcription and tissue-specific TF binding. CTCF-YY1 co-bound regions in liver tend to be associated with core liver functions such as lipid metabolism and transport in both human and mouse (Figure S6C in Additional file 1).

In order to determine whether the presence of YY1 at CTCF-bound regions has an effect on transcriptional output, the expression of genes bound by only CTCF versus those bound by CTCF and YY1 were compared. Genes overlapping YY1 binding events (including YY1-only and YY1-CTCF) are significantly more highly expressed than are genes overlapping CTCF-only binding events (*P* < 10^-16^) in both human and mouse liver (Figure 6C; Figure S6D in Additional file 1).

In sum, CTCF-YY1 co-bound regions are functionally similar to YY1-only regions based on their association with increased gene expression and enrichment of Pol II, H3K4me3 and liver-specific transcriptional regulators. This means that the CTCF occupied regions co-bound by YY1 show not only stronger evolutionary stability, but also increased transcriptional activity, in contrast to the bulk of CTCF-bound regions.

## Discussion

Our mapping and inter-species comparison of CTCF binding in cell lines from *H.sap*, *P.tro*, *G.gor*, *P.pyg*, *M.mul*, *P.ham* and *S.oed* has revealed over 11,000 genomic locations bound by CTCF across primates, consistent with the high conservation of CTCF binding observed in more distant mammalian species [19,21,67]. This estimate was obtained despite the fact that our analytical approach did not assume that CTCF binding is conserved, and as such is likely to underestimate true inter-species overlaps [21,22]. In contrast, other related studies have assumed conservation and minimized species-specific differences by using a dual cutoff, anchored in a single reference species [34,68].

Despite our conservative approach, we found that 60% of CTCF-bound regions are shared between *H.sap* and *M.mul*, whereas a recent comparison across 25 million years of *Drosophila* sp. evolution, a similar divergence time [69], has revealed approximately 30% binding conservation [70]. This discrepancy could be caused by differences in CTCF function between the chordate and arthropod phyla: CTCF is the only well-characterized insulator-binding protein in vertebrates, where it seems to be largely responsible for three-dimensional genome organization, as well as regulating various transcriptional and gene regulatory processes in collaboration with cohesin [71]. In contrast, *Drosophila* sp. have multiple insulator proteins and thus may place lower constraint on CTCF binding sites.

Previous studies have shown that the expansion of repetitive elements appears to be a major mechanism by which CTCF increases its target landscape in individual mammalian lineages [18,21,72]. Here, we identified particular repeat types associated with CTCF binding across primates, some of which have previously been shown to associate with CTCF in human, including LTR41 in human embryonic stem cells [18] and LTR13 across multiple cell types [18,63]. However, we did not find systematic evidence for a repeat-mediated expansion of CTCF binding in primate clades. This comparative quiescence of repeats that carry CTCF binding sequences in primates relative to rodents might be in part due to differences in genome transposon content and activity. For instance, mice have more lineage-specific repeat elements than humans, as well as greater transposon activity and fewer ancestral repeats [73,74].

## Conclusions

In searching for factors contributing to the conservation of CTCF binding we discovered that co-binding by YY1 is an ancient regulatory mechanism that appears to increase the evolutionary stability of CTCF binding in multiple mammalian species. CTCF and YY1 have previously been shown to co-localize and physically interact [9,42,43,46,75] but their combined, genome-wide interaction has not been investigated across multiple species.

Our analysis revealed that one mechanism stabilizing the protein-DNA contacts in regions co-bound by CTCF and YY1 may be their association with active chromatin and gene expression (previously reported for CTCF in [10,76-78]). It has been shown that CTCF can interact directly with Pol II and target it to a subset of CTCF sites genome-wide [79]. Our data now reveal that this likely occurs in the presence of YY1, because in its absence, CTCF-bound regions almost never co-localize with Pol II, H3K4me3, or tissue-specific TFs, whether proximal or distal to genes. These observations suggest that with respect to transcriptional activity, YY1 is the functionally dominant factor at co-bound locations.

Experiments performed at a single genetic locus have shown that CTCF can form a complex with YY1 and the tissue-specific factor Oct4 that binds *Tsix* and *Xite* to control X-chromosome pairing and counting in embryonic stem cells [80]. Co-binding of CTCF and YY1 thus appears to indicate globally to the chromatin remodeling machinery which euchromatic regions are to be activated [81]. The integration of these discoveries suggests a model wherein co-transcriptional activity of YY1-bound regions may help conserve CTCF and YY1 binding via functional deployment.

In summary, CTCF-YY1 co-bound regions are not only preferentially and highly conserved but also show hallmarks of transcriptional function that could provide selective pressure to preserve specific protein-DNA contacts across millions of years of mammalian evolution.

## Materials and methods

### Cell line material

Lymphoblast cell lines were obtained for seven primate species. The species, cell line and source are shown in Table S1 in Additional file 1. All cell lines were transformed by the Epstein-Barr virus except for the *M.mul* cell lines, which were transformed by Herpesvirus papio.

Cells were grown in suspension at a confluency of 200,000 cells/ml to 1 × 10^6^ cells/ml in RPMI1640 media supplemented with 15% fetal bovine serum and 2 mM L-glutamine, 100 I.U/ml penicillin and 100 μg/ml streptomycin. We cross-linked 1 × 10^8^ cells with 1% formaldehyde as previously described [82].

### Tissue material

#### Mouse

C57BL/6 J mice were housed in the Biological Resources Unit under UK Home Office licensing. Tissue was obtained from at least two independent males and formaldehyde cross-linked as described in [82].

#### Human

Male and female human tissue samples were obtained from biopsied tissue collected at Addenbrooke's Hospital, Cambridge, and provided by the Biobank under human tissue license 08/H0308/117. Liver tissue was also obtained from the Liver Tissue Distribution Program (NIDDK contract number N01-DK-9-2310) at the University of Pittsburgh.

#### ChIP-seq

ChIP-seq assays were performed as previously described [82]. Protein-bound DNA was immunoprecipitated with antibodies against CTCF (Millipore, Billerica, MA, USA, 07–729), or YY1 (Santa Cruz Biotechnology, Dallas, TX, USA, sc-281). Immunoprecipitated DNA was end-repaired, A-tailed and ligated to single-end Illumina sequencing adapters before 18 cycles of PCR amplification. DNA fragments (200 to 300 bp) were selected and 36 bp reads sequenced on an Illumina Genome Analyser II according to the manufacturer's instructions.

#### Published ChIP-seq experiments

The following published ChIP-seq data were used: mouse liver CTCF [21], mouse liver H3K4me3 and Pol II [83], human H3K4me3 and Pol II [66], human and mouse liver HNF4A and CEBPA [22]. ENCODE data used were from the following cell lines: for CTCF, GM12878, GM12891, GM12892, GM19239, GM19240, HepG2, H1-hESC; for YY1, GM12878, GM12891, GM12892, HepG2, H1-hESC; for NFKB, GM12878 (no treatment), GM12891 (tumor necrosis factor alpha treatment), GM12892 (tumor necrosis factor alpha treatment); for Pax5, GM12878, GM12891, GM12892; and for Pol II, GM12878 [43]. Published mouse embryonic stem cell ChIP-seq data for CTCF and YY1 were also used [19,84]).

### Computational methods

All computational analyses were performed with scripts written in Perl, Bioperl 1.2.3, and R version 2.11.1, using packages available in Bioconductor 2.6 (Additional file 2). Displayed error bars represent the standard error of the mean and significance levels were estimated using one-sided Wilcoxon rank-sum tests if not otherwise stated.

#### Read alignment and peak-calling

ChIP and input sequencing reads from all LCL datasets were aligned using Bowtie [85] 0.12.7 with the parameters '-n 2 -m 3 -k 1 -best' to the following genome assemblies: human GRCh37, chimpanzee CHIMP2.1, gorilla gorGor3, orangutan PPYG2, macaque Mmul 1, and marmoset C. jacchus 3.2.1. All sequence, genome annotations (genes, transcripts, CpG islands) and comparative genomics data were taken from Ensembl release 60. Repeat element annotation was downloaded from the UCSC Table Browser for all species. The baboon data were aligned to the macaque genome, as it was the closest fully assembled genome. When available (all species except for marmoset), only chromosomes and not unmapped contigs were used. Aligned reads were filtered for duplicates, uncalled bases (a maximum of three Ns were allowed) and low complexity reads. Regions of high ChIP enrichment (peaks/bound regions/binding events) were detected with CCAT 3.0 [86] on individual replicates using the parameters 'fragmentSize 100, slidingWinSize 150, movingStep 10, isStrandSensitiveMode 1, minCount 10, minScore 4.0, bootstrapPass 50' for the two TFs and 'fragmentSize 200, slidingWinSize 100, movingStep 20, isStrandSensitiveMode 0, minCount 10, minScore 4.0, bootstrapPass 50' for Pol II. Naked DNA (input) was used as control and the FDR cutoff was set to 0.1. Peaks were then merged among replicates in each organism by taking the intersection and additionally adding replicate-unique peaks with an FDR <0.05. The similarity between individual replicates was assessed by calculating the correlation (Spearman's rho) between read counts inside peak regions (Figure S1 in Additional file 1).

#### ChIP-seq data visualization

ChIP-seq data from each species was visualized on the corresponding species genome on the UCSC genome browser [87]. Human (GRCh37/hg19), chimpanzee (CGSC 2.1/panTro2), gorilla (gorGor3.1/gorGor3), orangutan (WUGSC 2.0.2/ponAbe2), macaque and baboon (MGSC Merged 1.0/rheMac2), tamarin (WUGSC 2.0.2/calJac1), and mouse (NCBI37/mm9).

#### Conservation analysis

We performed all our inter-species comparisons based on the 6-primate EPO (PrimateEPO) and the 11-way eutherian mammals (EPO) multiple sequence alignments (MSAs) available in Ensembl Compara release 60. Binding events discovered by CCAT at an FDR of 0.05 were projected onto all study species using the MSA through the Ensembl Compara Application Programming Interface (API). We restricted the evolutionary analysis to regions of the genome included in the MSA. Each of the study species was used as anchor species, and the region of interest projected onto the other species. In order to determine the degree of commonality between the species, projections were then overlapped (≥1 bp) with binding events - that is, binding events called at an FDR of 0.05 in one species that overlap with binding events called at the same FDR in a second species are called shared. To estimate the fraction of putatively shared binding events missed by this approach, we fixed the FDR in one species (human), varied it in the other six species (up to FDR = 0.5) and calculated the new percentage overlaps (data not shown). Conservation estimates increased by less than 10% compared to the fixed values reported in the manuscript, suggesting that while the method employed here does underestimate conservation levels, this effect is limited.

Overlap numbers differed by up to tens of bound regions depending on which species was used for anchoring. The percentage overlap numbers reported in Figure 2C, Figure S2B in Additional file 1, Figure 3D-E, and Figure S3D in Additional file 1 are averages between the two analysis directions (for example, shared human-chimpanzee regions from human and chimpanzee perspective). The human-human overlap percentage was obtained by calculating the overlap fraction of our operative peak set with different LCLs when available: five different cell lines for human (ENCODE LCL GM12878, GM12891, GM12892, GM19239, GM19239, GM19240), three different LCLs for chimpanzee and four different LCLs for rhesus macaque. The median value is displayed in Figure 2C based on primate (blue square) and mammalian (grey circle) alignments. Evolutionary time between the species was obtained from [88] (median).

For the comparative analyses displayed in Figures 2 and 3 and Figures S2 and S3 in Additional file 1 we divided the bound regions into six different categories in each of the seven primate species (we refer to these categories as 'conservation classes'): (i) species-specific and not included in the genome-wide multiple alignments, (ii) species-specific and included in the alignments, (iii) shared between two species only, (iv) shared among three to five species, (v) shared among six species, and (vi) shared among all seven analyzed primates. We calculated the relative fraction of bound regions belonging to these six categories and displayed them as barplots in Figure S2A in Additional file 1. The median values across all seven species are shown in Figure 2A as well as the number of seven-way shared peaks (based on the human genome).

For the sequence conservation analysis of CTCF-only and CTCF-YY1 regions in Figure S3E in Additional file 1, the Phastcons tool [89] in Galaxy Cistrome [90] was used.

#### Properties of different peak categories and CTCF-YY1 binding event classes

Four peak categories (species-specific (1), shared between exactly two species (2), shared among exactly six species (6) and shared among all seven species (7)) were further analyzed for diverse properties in each single species: CCAT score (proportional to ChIP enrichment), the top NestedMica motif match score distribution (with 0 corresponding to the consensus motif), the numbers of peaks with at least one motif, overlaps with peaks called in distinct LCL cell lines when available (human, two; chimpanzee, three; macaque, four), overlaps with transcripts, CpG islands, repetitive elements, Pol II, and publicly available TF binding data from ENCODE. Barplot widths are proportional to the number of regions belonging to each category. We also performed a detailed conservation-inter-individual overlap analysis using four distinct rhesus macaque LCL lines. We selected two types of CTCF-bound regions: (1) bound in only one of the four cell lines and (2) bound in all four cell lines. We then asked how often these regions were shared with the other species and displayed the relative fractions as pie charts in Figure 2D.

Three distinct classes of CTCF/YY1 bound regions (CTCF-only, CTCF-YY1 and YY1-only regions) were analyzed for their properties, using data from four primate LCLs (human, chimpanzee, orangutan, and baboon), as well as human and mouse liver data. Additionally, previously published Pol II, H3K4me3, CEBPA, and HNF4A data in human and mouse liver were intersected with the three classes of bound regions.

#### Repeat element association

We tested genome-wide association of annotated repeat elements with LCL CTCF/YY1 binding events in each single species by using a binomial test. We estimated background probabilities from median overlaps of repeat elements with randomized CTCF/YY1 binding events, and corrected for multiple testing by the Benjamini-Hochberg method. Repeats that obtained a *P*-value ≤0.01 are included in Figure S4A,D in Additional file 1; repeats with a -log *P*-value >10 in at least one species are displayed in Figure 4 and Table S3 in Additional file 1.

We estimated the repeat divergence from the consensus sequence based on the number of substitutions from the consensus ('milliDiv' column in the UCSC-obtained RepeatMasker tracks) and the age of individual bound repeat elements by dividing the substitution number by the mutation rate estimated for mammalian species (2.2 × 10^9^ per base pair per year) [91] and rodents (4.5 × 10^9^ per base pair per year) [74]. Repeat ages were used to order the heatmaps shown in Figure 4. Repeats were sorted by class in Figure S4 in Additional file 1[92]. Repeat profile plots centered on CTCF and YY1 peak summits in human LCLs were displayed for the top two enriched repeats, LTR13/LTR41, and MLT1J/MLT1F, respectively.

We also performed a detailed motif-word analysis as described in [21]. Individual motif instances obtained by scanning the genomes with the CTCF position weight matrix (PWM) were collected as DNA motif words (14-mers). We defined the set of bound words as the union of words falling inside bound regions in our study species. We counted individual occurrences of all motif words in the studied species, and divided by a normalization factor, proportional to the total number of bound bases in a certain species, obtaining a normalized occurrence (nocc) measure for each word and species:

nocci;j=nocci;j/factor

where nocc is the word count, i is the word number, j is the species number, and factor is defined as the total bound bases divided by 1,000,000. We selected only words that occurred at least five times in at least one species. We used these normalized word occurrence values to define species-specific words as follows:

$$\mathrm{normWord}=\log2\left(\left(\mathrm{nocc}\left(S\right)+1\right)=\max\left(\mathrm{nocc}\left(R\right)+1\right)\right)$$

where S is the species of interest and R all other species or all other species from a different branch of the evolutionary tree (considered groups were hominidae, Old World monkeys, New World monkeys, primates, and non-primate mammals). We fitted a normal distribution to normWord and chose a cutoff that corresponded to a FDR of 0.05 after multiple testing correction. All words with nocc(S) greater than the determined cutoff were selected for each species. For these selected words, we counted the number of CTCF-bound sequences of this type that are located inside annotated repeat elements. We display the log number of such words, as well as the analogous results obtained in mouse livers (for comparison) in Figure S4B in Additional file 1.

Repeat read profiles displayed in Figure 4E,F were generated by quantifying the read counts in 200 windows of 50 bp each centered around CTCF or YY1 peak summits that were contained in the repeat classes of interest. The obtained matrices were then visualized in Java TreeView while keeping the scale the same for each dataset [93].

#### Motif analysis

Motif discovery was conducted with NestedMica [94] using the parameters '-minLength 5 -maxLength 30 -numMotifs 6' and a fourth order background model trained on mammalian regulatory regions (DHS) data. Discovered motifs were confirmed using MEME [95], with the options '-nmotifs 5 -minsites 100 -minw 6 -maxw 25 -revcomp - maxsize 500000 -dna'. We selected the top 1,000 peaks ordered by CCAT score and used 25 bp up- and downstream of the peak summit as input for motif discovery. As the obtained top motifs were virtually identical in all studied species, we merged them into a single PWM that we used in further motif analysis steps. NestedMica's nmscan with a cutoff of −15 was used for motif matching (a score of 0 corresponds to a perfect match to the motif consensus) displayed in Figure S5C in Additional file 1 are obtained from all sequences that match the CTCF and YY1 PWMs inside regions positive for both CTCF and YY1 ChIP signal.

#### Functional association analysis

CTCF regions co-bound with YY1 were analyzed relative to all CTCF-bound regions in Figure S6C in Additional file 1 to determine whether these regions were associated with common biological pathways using cPath within the GREAT bioinformatic tool [96,97].

#### Expression analysis

We used published liver RNA-seq data in human and mouse to test the association between YY1/CTCF binding events with transcriptional activity [22]. Reads were mapped to Ensembl release 60 transcript annotation and transcript levels quantified using mmseq [98]. We compared log2(transcript estimates) for transcripts overlapping YY1-only, CTCF-only, or at least one CTCF-YY1 binding event using a Wilcoxon signed-rank test and display the data as boxplots in Figure 6C and Figure S6D in Additional file 1.

### Data access

CTCF and YY1 ChIP-seq data have been deposited under Arrayexpress, accession number E-MTAB-1511.

## Abbreviations

bp: base pair; C.jac: *Callithrix jacchus*; ChIP: chromatin immunoprecipitation; FDR: false discovery rate; G.gor: *Gorilla gorilla*; H.sap: *Homo sapiens*; LCL: lymphoblastoid cell line; M.mul: *Macaca mulatta*; MSA: multiple sequence alignment; P.ham: *Papio hamadryas*; P.pyg: *Pongo pygmaeus*; P.tro: *Pan troglodytes*; Pol II: RNA polymerase II; PWM: position weight matrix; S.oed: *Sanguinus oedipus*; TF: transcription factor.

## Competing interests

The authors declare that they have no competing interests.

## Authors' contributions

PCS, MCW, DTO, and PF designed and conceived experiments. PCS, MCW, and AJF analyzed data. MCW performed experiments. CEC cultured and cross-linked the macaque cell lines. PCS, MCW, DTO, and PF wrote the manuscript with input from all authors. YG, DTO, and PF oversaw the work. All authors read and approved the final manuscript.

## Supplementary Material

Additional file 1 **Supplementary Figures S1 to S6 and Tables S1 to S3. Figure S1.** CTCF ChIP-seq read correlations. **Figure S2.** properties of conserved and species-specific CTCF binding events. **Figure S3.** properties of CTCF and YY1 binding events. **Figure S4.** association of CTCF and YY1 binding events with repeats. **Figure S5.** characterization of CTCF-YY1 binding events. **Figure S6.** association of CTCF-YY1 binding events with marks of active transcription. **Table S1.** cell line sources. **Table S2.** ChIP-seq library summary. **Table S3.** CTCF and YY1 binding event repeat associations. Click here for file

Additional file 2 Scripts in Perl and R used for computational analyses. Click here for file

## References

[B1] Klenova EM Nicolas RH Paterson HF Carne AF Heath CM Goodwin GH Neiman PE Lobanenkov VV CTCF, a conserved nuclear factor required for optimal transcriptional activity of the chicken c-myc gene, is an 11-Zn-finger protein differentially expressed in multiple forms Mol Cell Biol 1993 14 7612 7624 8246978 10.1128/mcb.13.12.7612-7624.1993 PMC364833

[B2] Moon H Filippova G Loukinov D Pugacheva E Chen Q Smith ST Munhall A Grewe B Bartkuhn M Arnold R Burke LJ Renkawitz-Pohl R Ohlsson R Zhou J Renkawitz R Lobanenkov V CTCF is conserved from Drosophila to humans and confers enhancer blocking of the Fab-8 insulator EMBO Rep 2005 14 165 170 10.1038/sj.embor.7400334 15678159 PMC1299244

[B3] Baniahmad A Steiner C Kohne AC Renkawitz R Modular structure of a chicken lysozyme silencer: involvement of an unusual thyroid hormone receptor binding site Cell 1990 14 505 514 10.1016/0092-8674(90)90532-J 2159385

[B4] Lobanenkov VV Nicolas RH Adler VV Paterson H Klenova EM Polotskaja AV Goodwin GH A novel sequence-specific DNA binding protein which interacts with three regularly spaced direct repeats of the CCCTC-motif in the 5'-flanking sequence of the chicken c-myc gene Oncogene 1990 14 1743 1753 2284094

[B5] Vostrov AA Quitschke WW The zinc finger protein CTCF binds to the APBbeta domain of the amyloid beta-protein precursor promoter. Evidence for a role in transcriptional activation J Biol Chem 1997 14 33353 33359 10.1074/jbc.272.52.33353 9407128

[B6] Bell AC West AG Felsenfeld G The protein CTCF is required for the enhancer blocking activity of vertebrate insulators Cell 1999 14 387 396 10.1016/S0092-8674(00)81967-4 10458613

[B7] Hark AT Schoenherr CJ Katz DJ Ingram RS Levorse JM Tilghman SM CTCF mediates methylation-sensitive enhancer-blocking activity at the H19/Igf2 locus Nature 2000 14 486 489 10.1038/35013106 10839547

[B8] Shukla S Kavak E Gregory M Imashimizu M Shutinoski B Kashlev M Oberdoerffer P Sandberg R Oberdoerffer S CTCF-promoted RNA polymerase II pausing links DNA methylation to splicing Nature 2011 14 74 79 10.1038/nature10442 21964334 PMC7398428

[B9] Donohoe ME Zhang LF Xu N Shi Y Lee JT Identification of a Ctcf cofactor, Yy1, for the X chromosome binary switch Mol Cell 2007 14 43 56 10.1016/j.molcel.2006.11.017 17218270

[B10] Chen H Tian Y Shu W Bo X Wang S Comprehensive identification and annotation of cell type-specific and ubiquitous CTCF-binding sites in the human genome PLoS One 2012 14 e41374 10.1371/journal.pone.0041374 22829947 PMC3400636

[B11] Cuddapah S Jothi R Schones DE Roh TY Cui K Zhao K Global analysis of the insulator binding protein CTCF in chromatin barrier regions reveals demarcation of active and repressive domains Genome Res 2009 14 24 32 19056695 10.1101/gr.082800.108 PMC2612964

[B12] Kim TH Abdullaev ZK Smith AD Ching KA Loukinov DI Green RD Zhang MQ Lobanenkov VV Ren B Analysis of the vertebrate insulator protein CTCF-binding sites in the human genome Cell 2007 14 1231 1245 10.1016/j.cell.2006.12.048 17382889 PMC2572726

[B13] Fu Y Sinha M Peterson CL Weng Z The insulator binding protein CTCF positions 20 nucleosomes around its binding sites across the human genome PLoS Genet 2008 14 e1000138 10.1371/journal.pgen.1000138 18654629 PMC2453330

[B14] Handoko L Xu H Li G Ngan CY Chew E Schnapp M Lee CW Ye C Ping JL Mulawadi F Wong E Sheng J Zhang Y Poh T Chan CS Kunarso G Shahab A Bourque G Cacheux-Rataboul V Sung WK Ruan Y Wei CL CTCF-mediated functional chromatin interactome in pluripotent cells Nat Genet 2011 14 630 638 10.1038/ng.857 21685913 PMC3436933

[B15] Wang H Maurano MT Qu H Varley KE Gertz J Pauli F Lee K Canfield T Weaver M Sandstrom R Thurman RE Kaul R Myers RM Stamatoyannopoulos JA Widespread plasticity in CTCF occupancy linked to DNA methylation Genome Res 2012 14 1680 1688 10.1101/gr.136101.111 22955980 PMC3431485

[B16] Shen Y Yue F McCleary DF Ye Z Edsall L Kuan S Wagner U Dixon J Lee L Lobanenkov VV Ren B A map of the cis-regulatory sequences in the mouse genome Nature 2012 14 116 120 10.1038/nature11243 22763441 PMC4041622

[B17] Borneman AR Gianoulis TA Zhang ZD Yu H Rozowsky J Seringhaus MR Wang LY Gerstein M Snyder M Divergence of transcription factor binding sites across related yeast species Science 2007 14 815 819 10.1126/science.1140748 17690298

[B18] Kunarso G Chia NY Jeyakani J Hwang C Lu X Chan YS Ng HH Bourque G Transposable elements have rewired the core regulatory network of human embryonic stem cells Nat Genet 2010 14 631 634 10.1038/ng.600 20526341

[B19] Martin D Pantoja C Fernandez Minan A Valdes-Quezada C Molto E Matesanz F Bogdanovic O de la Calle-Mustienes E Dominguez O Taher L Furlan-Magaril M Alcina A Canon S Fedetz M Blasco MA Pereira PS Ovcharenko I Recillas-Targa F Montoliu L Manzanares M Guigo R Serrano M Casares F Gomez-Skarmeta JL Genome-wide CTCF distribution in vertebrates defines equivalent sites that aid the identification of disease-associated genes Nat Struct Mol Biol 2011 14 708 714 10.1038/nsmb.2059 21602820 PMC3196567

[B20] Odom DT Dowell RD Jacobsen ES Gordon W Danford TW MacIsaac KD Rolfe PA Conboy CM Gifford DK Fraenkel E Tissue-specific transcriptional regulation has diverged significantly between human and mouse Nat Genet 2007 14 730 732 10.1038/ng2047 17529977 PMC3797512

[B21] Schmidt D Schwalie PC Wilson MD Ballester B Goncalves A Kutter C Brown GD Marshall A Flicek P Odom DT Waves of retrotransposon expansion remodel genome organization and CTCF binding in multiple mammalian lineages Cell 2012 14 335 348 10.1016/j.cell.2011.11.058 22244452 PMC3368268

[B22] Schmidt D Wilson MD Ballester B Schwalie PC Brown GD Marshall A Kutter C Watt S Martinez-Jimenez CP Mackay S Talianidis I Flicek P Odom DT Five-vertebrate ChIP-seq reveals the evolutionary dynamics of transcription factor binding Science 2010 14 1036 1040 10.1126/science.1186176 20378774 PMC3008766

[B23] CSAC Initial sequence of the chimpanzee genome and comparison with the human genome Nature 2005 14 69 87 10.1038/nature04072 16136131

[B24] Gibbs RA Rogers J Katze MG Bumgarner R Weinstock GM Mardis ER Remington KA Strausberg RL Venter JC Wilson RK Batzer MA Bustamante CD Eichler EE Hahn MW Hardison RC Makova KD Miller W Milosavljevic A Palermo RE Siepel A Sikela JM Attaway T Bell S Bernard KE Buhay CJ Chandrabose MN Dao M Davis C Delehaunty KD Ding Y Evolutionary and biomedical insights from the rhesus macaque genome Science 2007 14 222 234 17431167 10.1126/science.1139247

[B25] Locke DP Hillier LW Warren WC Worley KC Nazareth LV Muzny DM Yang SP Wang Z Chinwalla AT Minx P Mitreva M Cook L Delehaunty KD Fronick C Schmidt H Fulton LA Fulton RS Nelson JO Magrini V Pohl C Graves TA Markovic C Cree A Dinh HH Hume J Kovar CL Fowler GR Lunter G Meader S Heger A Comparative and demographic analysis of orang-utan genomes Nature 2011 14 529 533 10.1038/nature09687 21270892 PMC3060778

[B26] Scally A Dutheil JY Hillier LW Jordan GE Goodhead I Herrero J Hobolth A Lappalainen T Mailund T Marques-Bonet T McCarthy S Montgomery SH Schwalie PC Tang YA Ward MC Xue Y Yngvadottir B Alkan C Andersen LN Ayub Q Ball EV Beal K Bradley BJ Chen Y Clee CM Fitzgerald S Graves TA Gu Y Heath P Heger A Insights into hominid evolution from the gorilla genome sequence Nature 2012 14 169 175 10.1038/nature10842 22398555 PMC3303130

[B27] Sugimoto M Tahara H Ide T Furuichi Y Steps involved in immortalization and tumorigenesis in human B-lymphoblastoid cell lines transformed by Epstein-Barr virus Cancer Res 2004 14 3361 3364 10.1158/0008-5472.CAN-04-0079 15150084

[B28] Barreiro LB Marioni JC Blekhman R Stephens M Gilad Y Functional comparison of innate immune signaling pathways in primates PLoS Genet 2010 14 e1001249 10.1371/journal.pgen.1001249 21187902 PMC3002988

[B29] Blekhman R Oshlack A Chabot AE Smyth GK Gilad Y Gene regulation in primates evolves under tissue-specific selection pressures PLoS Genet 2008 14 e1000271 10.1371/journal.pgen.1000271 19023414 PMC2581600

[B30] Brawand D Soumillon M Necsulea A Julien P Csardi G Harrigan P Weier M Liechti A Aximu-Petri A Kircher M Albert FW Zeller U Khaitovich P Grutzner F Bergmann S Nielsen R Paabo S Kaessmann H The evolution of gene expression levels in mammalian organs Nature 2011 14 343 348 10.1038/nature10532 22012392

[B31] Khaitovich P Enard W Lachmann M Paabo S Evolution of primate gene expression Nat Rev Genet 2006 14 693 702 10.1038/nrg1940 16921347

[B32] Perry GH Melsted P Marioni JC Wang Y Bainer R Pickrell JK Michelini K Zehr S Yoder AD Stephens M Pritchard JK Gilad Y Comparative RNA sequencing reveals substantial genetic variation in endangered primates Genome Res 2011 14 602 610 22207615 10.1101/gr.130468.111 PMC3317143

[B33] King MC Wilson AC Evolution at two levels in humans and chimpanzees Science 1975 14 107 116 10.1126/science.1090005 1090005

[B34] Cain CE Blekhman R Marioni JC Gilad Y Gene expression differences among primates are associated with changes in a histone epigenetic modification Genetics 2011 14 1225 1234 10.1534/genetics.110.126177 21321133 PMC3070530

[B35] Martin DI Singer M Dhahbi J Mao G Zhang L Schroth GP Pachter L Boffelli D Phyloepigenomic comparison of great apes reveals a correlation between somatic and germline methylation states Genome Res 2011 14 2049 2057 10.1101/gr.122721.111 21908772 PMC3227095

[B36] Pai AA Bell JT Marioni JC Pritchard JK Gilad Y A genome-wide study of DNA methylation patterns and gene expression levels in multiple human and chimpanzee tissues PLoS Genet 2011 14 e1001316 10.1371/journal.pgen.1001316 21383968 PMC3044686

[B37] Shibata Y Sheffield NC Fedrigo O Babbitt CC Wortham M Tewari AK London D Song L Lee BK Iyer VR Partker SC Margulies EH Wray EH Furey TS Crawford GE Extensive evolutionary changes in regulatory element activity during human origins are associated with altered gene expression and positive selection PLoS Genet 2012 14 e1002789 10.1371/journal.pgen.1002789 22761590 PMC3386175

[B38] Chabot A Shrit RA Blekhman R Gilad Y Using reporter gene assays to identify cis regulatory differences between humans and chimpanzees Genetics 2007 14 2069 2076 10.1534/genetics.107.073429 17565944 PMC1950614

[B39] Iskow RC Gokcumen O Abyzov A Malukiewicz J Zhu Q Sukumar AT Pai AA Mills RE Habegger L Cusanovich DA Rubel MA Perry GH Gerstein M Stone AC Gilad Y Lee C Regulatory element copy number differences shape primate expression profiles Proc Natl Acad Sci U S A 2012 14 12656 12661 10.1073/pnas.1205199109 22797897 PMC3411951

[B40] Weth O Renkawitz R CTCF function is modulated by neighboring DNA binding factors Biochem Cell Biol 2011 14 459 468 10.1139/o11-033 21895576

[B41] Zlatanova J Caiafa P CTCF and its protein partners: divide and rule? J Cell Sci 2009 14 1275 1284 10.1242/jcs.039990 19386894

[B42] Wang J Lunyak VV Jordan IK Genome-wide prediction and analysis of human chromatin boundary elements Nucleic Acids Res 2011 14 511 529 21930510 10.1093/nar/gkr750 PMC3258141

[B43] Dunham I Kundaje A Aldred SF Collins PJ Davis CA Doyle F Epstein CB Frietze S Harrow J Kaul R Khatun J Lajoie BR Landt SG Lee BK Pauli F Rosenbloom KR Sabo P Safi A Sanyal A Shoresh N Simon JM Song L Trinklein ND Altshuler RC Birney E Brown JB Cheng C Djebali S Dong X Dunham I An integrated encyclopedia of DNA elements in the human genome Nature 2012 14 57 74 10.1038/nature11247 22955616 PMC3439153

[B44] Guo Y Mahony S Gifford DK High resolution genome wide binding event finding and motif discovery reveals transcription factor spatial binding constraints PLoS Comput Biol 2012 14 e1002638 10.1371/journal.pcbi.1002638 22912568 PMC3415389

[B45] Shi Y Seto E Chang LS Shenk T Transcriptional repression by YY1, a human GLI-Kruppel-related protein, and relief of repression by adenovirus E1A protein Cell 1991 14 377 388 10.1016/0092-8674(91)90189-6 1655281

[B46] Kim J Kim JD In vivo YY1 knockdown effects on genomic imprinting Hum Mol Genet 2008 14 391 401 17977899 10.1093/hmg/ddm316

[B47] Kim JD Hinz AK Bergmann A Huang JM Ovcharenko I Stubbs L Kim J Identification of clustered YY1 binding sites in imprinting control regions Genome Res 2006 14 901 911 10.1101/gr.5091406 16760423 PMC1484457

[B48] Kim JD Hinz AK Choo JH Stubbs L Kim J YY1 as a controlling factor for the Peg3 and Gnas imprinted domains Genomics 2007 14 262 269 10.1016/j.ygeno.2006.09.009 17067777 PMC1828871

[B49] Jeon Y Lee JT YY1 tethers Xist RNA to the inactive X nucleation center Cell 2011 14 119 133 10.1016/j.cell.2011.06.026 21729784 PMC3150513

[B50] Wu S Hu YC Liu H Shi Y Loss of YY1 impacts the heterochromatic state and meiotic double-strand breaks during mouse spermatogenesis Mol Cell Biol 2009 14 6245 6256 10.1128/MCB.00679-09 19786570 PMC2786691

[B51] Donohoe ME Zhang X McGinnis L Biggers J Li E Shi Y Targeted disruption of mouse Yin Yang 1 transcription factor results in peri-implantation lethality Mol Cell Biol 1999 14 7237 7244 10490658 10.1128/mcb.19.10.7237 PMC84716

[B52] Atchison L Ghias A Wilkinson F Bonini N Atchison ML Transcription factor YY1 functions as a PcG protein in vivo EMBO J 2003 14 1347 1358 10.1093/emboj/cdg124 12628927 PMC151054

[B53] Brown JL Mucci D Whiteley M Dirksen ML Kassis JA The Drosophila Polycomb group gene pleiohomeotic encodes a DNA binding protein with homology to the transcription factor YY1 Mol Cell 1998 14 1057 1064 10.1016/S1097-2765(00)80106-9 9651589

[B54] Xi H Yu Y Fu Y Foley J Halees A Weng Z Analysis of overrepresented motifs in human core promoters reveals dual regulatory roles of YY1 Genome Res 2007 14 798 806 10.1101/gr.5754707 17567998 PMC1891339

[B55] Schug J Schuller WP Kappen C Salbaum JM Bucan M Stoeckert CJ Jr Promoter features related to tissue specificity as measured by Shannon entropy Genome Biol 2005 14 R33 10.1186/gb-2005-6-4-r33 15833120 PMC1088961

[B56] Athanikar JN Badge RM Moran JV A YY1-binding site is required for accurate human LINE-1 transcription initiation Nucleic Acids Res 2004 14 3846 3855 10.1093/nar/gkh698 15272086 PMC506791

[B57] Becker KG Swergold GD Ozato K Thayer RE Binding of the ubiquitous nuclear transcription factor YY1 to a cis regulatory sequence in the human LINE-1 transposable element Hum Mol Genet 1993 14 1697 1702 10.1093/hmg/2.10.1697 8268924

[B58] Humphrey GW Englander EW Howard BH Specific binding sites for a pol III transcriptional repressor and pol II transcription factor YY1 within the internucleosomal spacer region in primate Alu repetitive elements Gene Expr 1996 14 151 168 9041122 PMC6148310

[B59] Knossl M Lower R Lower J Expression of the human endogenous retrovirus HTDV/HERV-K is enhanced by cellular transcription factor YY1 J Virol 1999 14 1254 1261 9882329 10.1128/jvi.73.2.1254-1261.1999 PMC103948

[B60] Satyamoorthy K Park K Atchison ML Howe CC The intracisternal A-particle upstream element interacts with transcription factor YY1 to activate transcription: pleiotropic effects of YY1 on distinct DNA promoter elements Mol Cell Biol 1993 14 6621 6628 8413258 10.1128/mcb.13.11.6621-6628.1993 PMC364725

[B61] Flicek P Amode MR Barrell D Beal K Brent S Carvalho-Silva D Clapham P Coates G Fairley S Fitzgerald S Ensembl 2012 Nucleic Acids Res 2012 14 D84 D90 10.1093/nar/gkr991 22086963 PMC3245178

[B62] Stefflova K Thybert T Wilson MD Streeter I Aleksic J Karagianni P Talianidis I Brazma A Adams D Marioni J Cooperativity and rapid evolution of co-bound transcription factors in closely related mammals Cell 2013 14 530 540 10.1016/j.cell.2013.07.007 23911320 PMC3732390

[B63] Jacques PE Jeyakani J Bourque G The majority of primate-specific regulatory sequences are derived from transposable elements PLoS Genet 2013 14 e1003504 10.1371/journal.pgen.1003504 23675311 PMC3649963

[B64] Liao D Pavelitz T Weiner AM Characterization of a novel class of interspersed LTR elements in primate genomes: structure, genomic distribution, and evolution J Mol Evol 1998 14 649 660 10.1007/PL00006345 9608047

[B65] Cohen CJ Lock WM Mager DL Endogenous retroviral LTRs as promoters for human genes: a critical assessment Gene 2009 14 105 114 10.1016/j.gene.2009.06.020 19577618

[B66] Ward MC Wilson MD Barbosa-Morais NL Schmidt D Stark R Pan Q Schwalie PC Menon S Lukk M Watt S Thybert D Kutter C Kirschner K Flicek P Blencowe BJ Odom DT Latent regulatory potential of human-specific repetitive elements Mol Cell 2013 14 262 272 10.1016/j.molcel.2012.11.013 23246434 PMC3560060

[B67] Xie X Mikkelsen TS Gnirke A Lindblad-Toh K Kellis M Lander ES Systematic discovery of regulatory motifs in conserved regions of the human genome, including thousands of CTCF insulator sites Proc Natl Acad Sci U S A 2007 14 7145 7150 10.1073/pnas.0701811104 17442748 PMC1852749

[B68] He Q Bardet AF Patton B Purvis J Johnston J Paulson A Gogol M Stark A Zeitlinger J High conservation of transcription factor binding and evidence for combinatorial regulation across six Drosophila species Nat Genet 2011 14 414 420 10.1038/ng.808 21478888

[B69] Tamura K Subramanian S Kumar S Temporal patterns of fruit fly (Drosophila) evolution revealed by mutation clocks Mol Biol Evol 2004 14 36 44 12949132 10.1093/molbev/msg236

[B70] Ni X Zhang YE Negre N Chen S Long M White KP Adaptive evolution and the birth of CTCF binding sites in the Drosophila genome PLoS Biol 2012 14 e1001420 10.1371/journal.pbio.1001420 23139640 PMC3491045

[B71] Merkenschlager M Odom DT CTCF and cohesin: linking gene regulatory elements with their targets Cell 2013 14 1285 1297 10.1016/j.cell.2013.02.029 23498937

[B72] Bourque G Leong B Vega VB Chen X Lee YL Srinivasan KG Chew JL Ruan Y Wei CL Ng HH Liu ET Evolution of the mammalian transcription factor binding repertoire via transposable elements Genome Res 2008 14 1752 1762 10.1101/gr.080663.108 18682548 PMC2577865

[B73] Maksakova IA Romanish MT Gagnier L Dunn CA van de Lagemaat LN Mager DL Retroviral elements and their hosts: insertional mutagenesis in the mouse germ line PLoS Genet 2006 14 e2 10.1371/journal.pgen.0020002 16440055 PMC1331978

[B74] Waterston RH Lindblad-Toh K Birney E Rogers J Abril JF Agarwal P Agarwala R Ainscough R Alexandersson M An P Antonarakis SE Attwood J Baertsch R Bailey J Barlow K Beck S Berry E Birren B Bloom T Bork P Botcherby M Bray N Brent MR Brown DG Brown SD Bult C Burton J Butler J Campbell RD Carninci P Initial sequencing and comparative analysis of the mouse genome Nature 2002 14 520 562 10.1038/nature01262 12466850

[B75] Kang K Chung JH Kim J Evolutionary Conserved Motif Finder (ECMFinder) for genome-wide identification of clustered YY1- and CTCF-binding sites Nucleic Acids Res 2009 14 2003 2013 10.1093/nar/gkp077 19208640 PMC2665242

[B76] Essien K Vigneau S Apreleva S Singh LN Bartolomei MS Hannenhalli S CTCF binding site classes exhibit distinct evolutionary, genomic, epigenomic and transcriptomic features Genome Biol 2009 14 R131 10.1186/gb-2009-10-11-r131 19922652 PMC3091324

[B77] Negre N Brown CD Ma L Bristow CA Miller SW Wagner U Kheradpour P Eaton ML Loriaux P Sealfon R Li Z Ishii H Spokony RF Chen J Hwang L Cheng C Auburn RP Davis MB Domanus M Shah PK Morrison CA Zieba J Suchy S Senderowicz L Victorsen A Bild NA Grundstad AJ Hanley D MacAlpine DM Mannervik M A cis-regulatory map of the Drosophila genome Nature 2011 14 527 531 10.1038/nature09990 21430782 PMC3179250

[B78] Rach EA Winter DR Benjamin AM Corcoran DL Ni T Zhu J Ohler U Transcription initiation patterns indicate divergent strategies for gene regulation at the chromatin level PLoS Genet 2011 14 e1001274 10.1371/journal.pgen.1001274 21249180 PMC3020932

[B79] Chernukhin I Shamsuddin S Kang SY Bergstrom R Kwon YW Yu W Whitehead J Mukhopadhyay R Docquier F Farrar D Morrison I Vigneron M Wu SY Chiang CM Loukinov D Lobanenkov V Ohlsson R Klenova E CTCF interacts with and recruits the largest subunit of RNA polymerase II to CTCF target sites genome-wide Mol Cell Biol 2007 14 1631 1648 10.1128/MCB.01993-06 17210645 PMC1820452

[B80] Donohoe ME Silva SS Pinter SF Xu N Lee JT The pluripotency factor Oct4 interacts with Ctcf and also controls X-chromosome pairing and counting Nature 2009 14 128 132 10.1038/nature08098 19536159 PMC3057664

[B81] Cai Y Jin J Yao T Gottschalk AJ Swanson SK Wu S Shi Y Washburn MP Florens L Conaway RC Conaway JW YY1 functions with INO80 to activate transcription Nat Struct Mol Biol 2007 14 872 874 10.1038/nsmb1276 17721549

[B82] Schmidt D Wilson MD Spyrou C Brown GD Hadfield J Odom DT ChIP-seq: using high-throughput sequencing to discover protein-DNA interactions Methods 2009 14 240 248 10.1016/j.ymeth.2009.03.001 19275939 PMC4052679

[B83] Vella P Barozzi I Cuomo A Bonaldi T Pasini D Yin Yang 1 extends the Myc-related transcription factors network in embryonic stem cells Nucleic Acids Res 2012 14 3403 3418 10.1093/nar/gkr1290 22210892 PMC3333890

[B84] Faure AJ Schmidt D Watt S Schwalie PC Wilson MD Xu H Ramsay RG Odom DT Flicek P Cohesin regulates tissue-specific expression by stabilizing highly occupied cis-regulatory modules Genome Res 2012 14 2163 2175 10.1101/gr.136507.111 22780989 PMC3483546

[B85] Langmead B Aligning short sequencing reads with Bowtie Curr Protoc Bioinformatics 2010 14 Unit 11.7 21154709 10.1002/0471250953.bi1107s32 PMC3010897

[B86] Xu H Handoko L Wei X Ye C Sheng J Wei CL Lin F Sung WK A signal-noise model for significance analysis of ChIP-seq with negative control Bioinformatics 2010 14 1199 1204 10.1093/bioinformatics/btq128 20371496

[B87] Kent WJ Sugnet CW Furey TS Roskin KM Pringle TH Zahler AM Haussler D The human genome browser at UCSC Genome Res 2002 14 996 1006 10.1101/gr.229102 12045153 PMC186604

[B88] Hedges SB Dudley J Kumar S TimeTree: a public knowledge-base of divergence times among organisms Bioinformatics 2006 14 2971 2972 10.1093/bioinformatics/btl505 17021158

[B89] Siepel A Bejerano G Pedersen JS Hinrichs AS Hou M Rosenbloom K Clawson H Spieth J Hillier LW Richards S Weinstock GM Wilson RK Gibbs RA Kent WJ Miller W Haussler D Evolutionarily conserved elements in vertebrate, insect, worm, and yeast genomes Genome Res 2005 14 1034 1050 10.1101/gr.3715005 16024819 PMC1182216

[B90] Liu T Ortiz JA Taing L Meyer CA Lee B Zhang Y Shin H Wong SS Ma J Lei Y Pape UJ Poidinger M Chen Y Yeung K Brown M Turpaz Y Liu XS Cistrome: an integrative platform for transcriptional regulation studies Genome Biol 2011 14 R83 10.1186/gb-2011-12-8-r83 21859476 PMC3245621

[B91] Kumar S Subramanian S Mutation rates in mammalian genomes Proc Natl Acad Sci U S A 2002 14 803 808 10.1073/pnas.022629899 11792858 PMC117386

[B92] Jurka J Kapitonov VV Pavlicek A Klonowski P Kohany O Walichiewicz J Repbase Update, a database of eukaryotic repetitive elements Cytogenet Genome Res 2005 14 462 467 10.1159/000084979 16093699

[B93] Saldanha AJ Java Treeview–extensible visualization of microarray data Bioinformatics 2004 14 3246 3248 10.1093/bioinformatics/bth349 15180930

[B94] Down TA Hubbard TJ NestedMICA: sensitive inference of over-represented motifs in nucleic acid sequence Nucleic Acids Res 2005 14 1445 1453 10.1093/nar/gki282 15760844 PMC1064142

[B95] Bailey TL Boden M Buske FA Frith M Grant CE Clementi L Ren J Li WW Noble WS MEME SUITE: tools for motif discovery and searching Nucleic Acids Res 2009 14 W202 W208 10.1093/nar/gkp335 19458158 PMC2703892

[B96] Cerami EG Bader GD Gross BE Sander C cPath: open source software for collecting, storing, and querying biological pathways BMC Bioinformatics 2006 14 497 10.1186/1471-2105-7-497 17101041 PMC1660554

[B97] McLean CY Bristor D Hiller M Clarke SL Schaar BT Lowe CB Wenger AM Bejerano G GREAT improves functional interpretation of cis-regulatory regions Nat Biotechnol 2010 14 495 501 10.1038/nbt.1630 20436461 PMC4840234

[B98] Turro E Su SY Gonçalves Â Coin LJ Richardson S Lewin A Haplotype and isoform specific expression estimation using multi-mapping RNA-seq reads Genome Biol 2011 14 R13 10.1186/gb-2011-12-2-r13 21310039 PMC3188795

