# Supplementary material for: Co-binding by YY1 identifies the transcriptionally active, highly conserved set of CTCF-bound regions in primate genomes
Source: Genome Biol. 2013 Dec 31;14(12):R148. doi: 10.1186/gb-2013-14-12-r148 (PMC4056453; doi:10.1186/gb-2013-14-12-r148)
Supplement: Additional file 1 — Supplementary Figures S1 to S6 and Tables S1 to S3. Figure S1. CTCF ChIP-seq read correlations. Figure S2. properties of conserved and species-specific CTCF binding events. Figure S3. properties of CTCF and YY1 binding events. Figure S4. association of CTCF and YY1 binding events with repeats. Figure S5. characterization of CTCF-YY1 binding events. Figure S6. association of CTCF-YY1 binding events with marks of active transcription. Table S1. cell line sources. Table S2. ChIP-seq library summary. Table S3. CTCF and YY1 binding event repeat associations. [file gb-2013-14-12-r148-S1.doc]

**TABLE OF CONTENTS**

SUPPLEMENTAL FIGURES

Figure S1: CTCF ChIP-seq peak correlations

Figure S2: Properties of conserved and species-specific CTCF binding events

Figure S3: Properties of CTCF and YY1 binding events

Figure S4: Association of CTCF and YY1 binding events with repeats

Figure S5: Characterization of CTCF-YY1 binding events

Figure S6: Association of CTCF-YY1 binding events with marks of active transcription

SUPPLEMENTAL TABLES

Table S1: Cell line sources

Table S2: ChIP-seq library summary

Table S3: CTCF and YY1 binding event repeat associations

SUPPLEMENTAL FIGURES

**
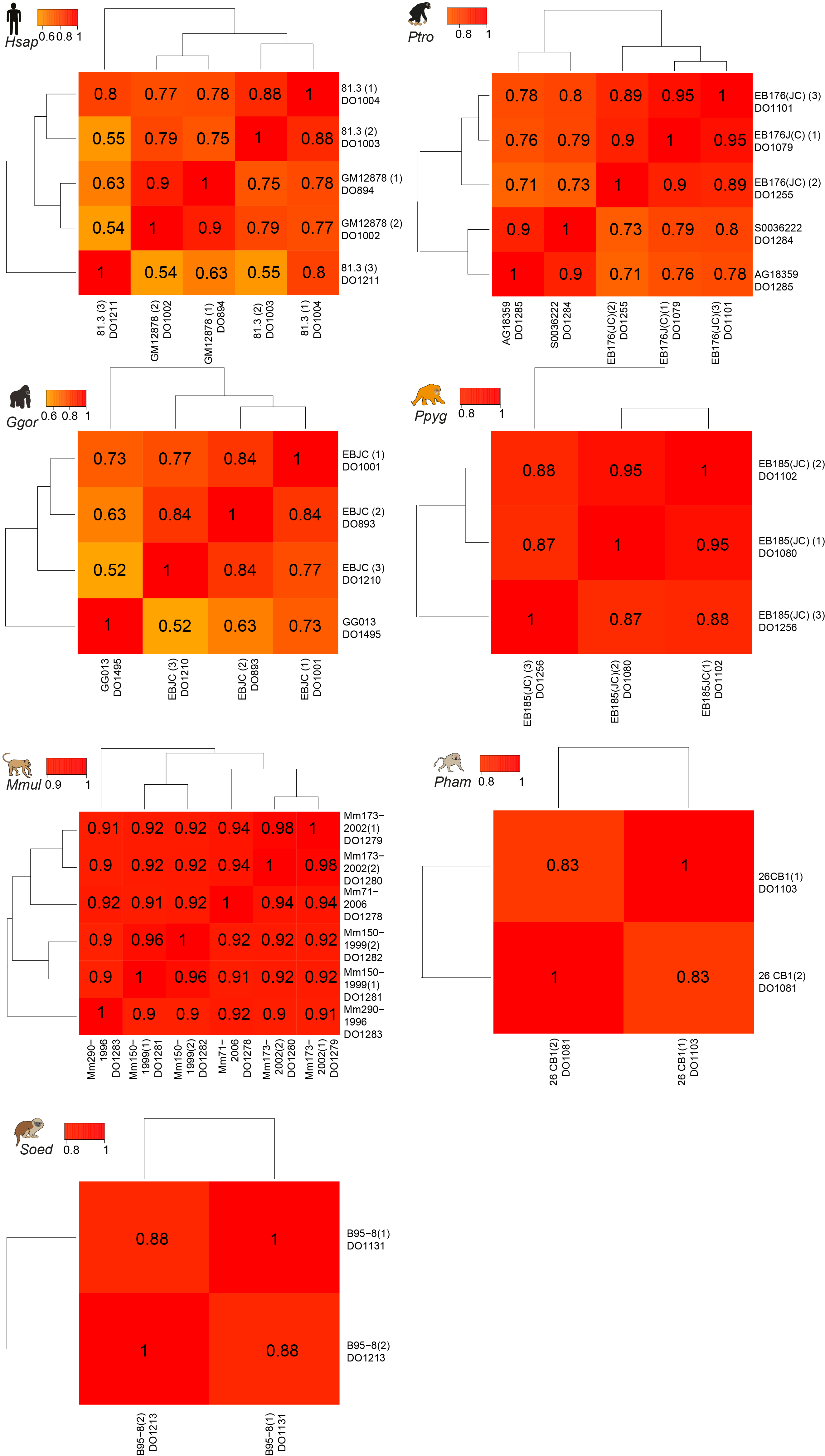
**

**Figure S1: Technical and biological CTCF ChIP-seq replicates correlate within a species.** Spearman correlation values between CTCF read counts (at peak regions) are shown in each species and visualized as a hierarchically clustered heatmap.

**
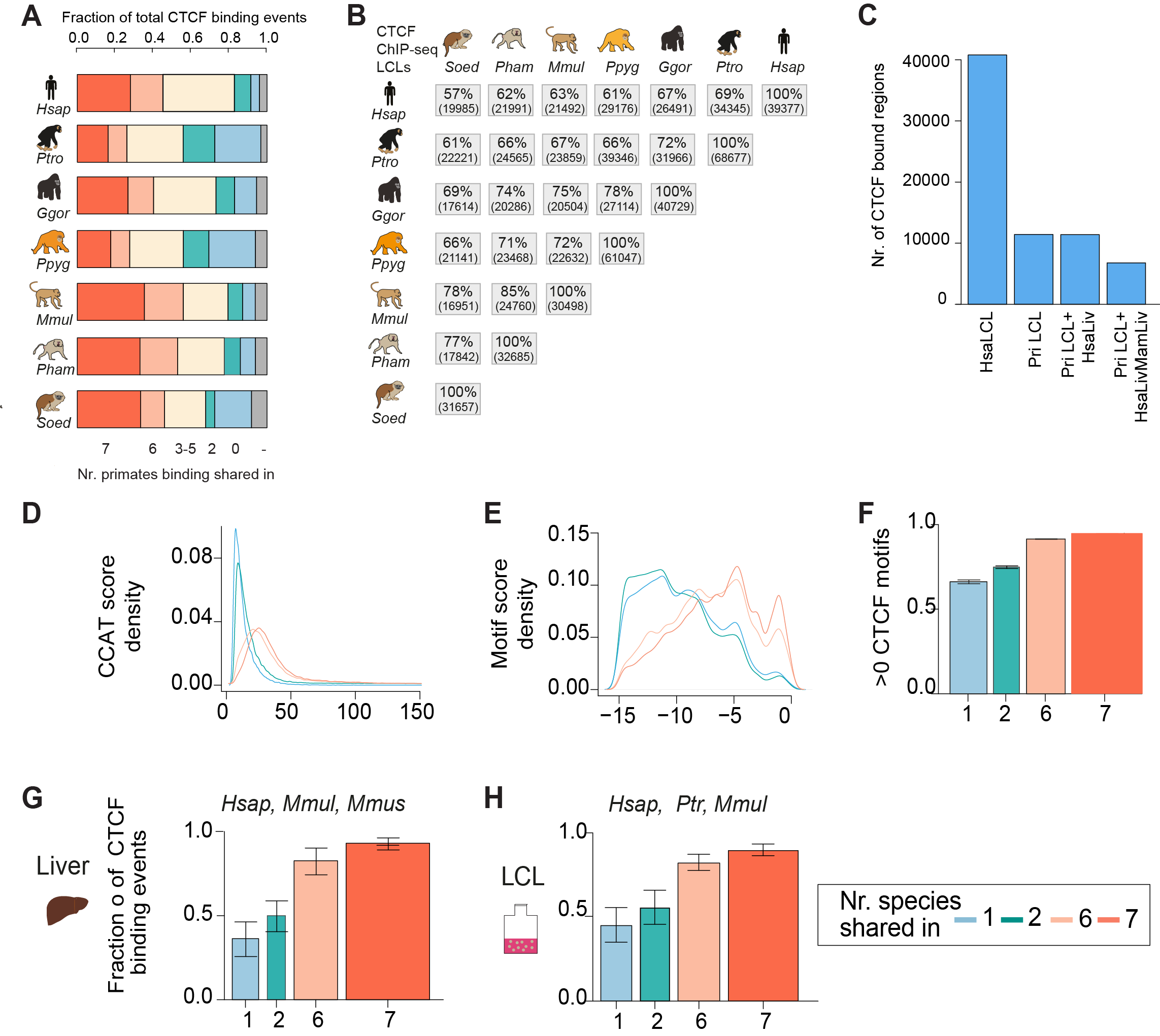
**

**Figure S2: CTCF binding events are highly conserved. A)** Fraction of total CTCF binding events in each conservation category for each individual species. **B)** Pairwise CTCF ChIP-seq interspecies overlaps represented as percentages. Total number of overlapping CTCF binding events are indicated in parentheses. **C)** Total number of CTCF binding events in *H.sap* LCLs, *H.sap* LCL CTCF binding events shared with all other primate LCLs, and with *H.sap* and *M.mus* liver. **D)** Density of CCAT peak caller scores across different conservation categories of CTCF binding events. **E)** Density of NestedMica's nmscan scores across different conservation categories of CTCF binding events (0 corresponds to a perfect motif match to the consensus). **F)** Fraction of CTCF binding events containing at least one CTCF binding motif within each of the conservation categories. **G)** Fraction of CTCF binding events within each conservation category averaged across *H.sap*, *M.mul* and *M.mus* liver. **H)** Fraction of CTCF binding events within each conservation category averaged across *H.sap*, *P.tro* and *M.mul* LCLs. (*p < 0.05, Wilcoxon rank-sum test, 1|2 vs. 6|7). Error bars represent the standard error of the mean among seven species in F and three species in G and H (*p <0.05, Wilcoxon rank-sum test, C vs. C+Y).


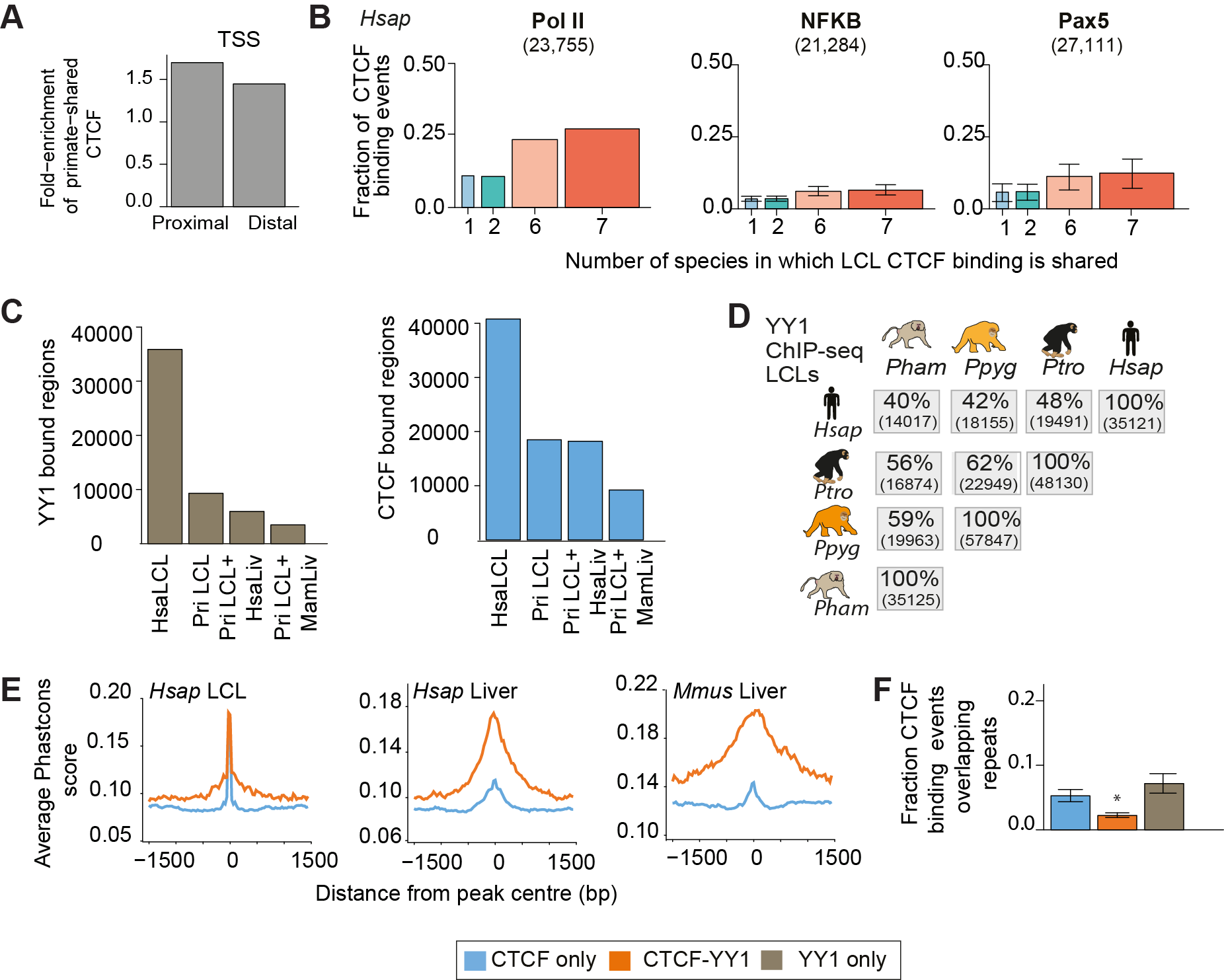


**Figure S3: Evolutionarily conserved CTCF binding events are associated with YY1. A)** Fold enrichment of primate-shared CTCF sites in CTCF-YY1 compared to non-YY1 regions located proximal (within 500 bp) and distal to the TSS. **B)** Fraction of CTCF binding events in each conservation category associated with Pol II, NFKB, and Pax5 in *H.sap* LCLs (ENCODE, Dunham et al., 2012). **C)** Total number of YY1 and CTCF binding events in *H.sap* LCLs; binding events shared with *H.sap*, *P.tro*, *P.pyg* and *P.ham* LCLs as well as *H.sap* and *M.mus* liver. **D)** Pairwise YY1 ChIP-seq interspecies overlaps represented as percentages. Total number of overlapping YY1 binding events are indicated in parentheses. **E)** Phastcons score of CTCF-only and CTCF-YY1 bound regions in *H.sap* LCLs, *H.sap* liver and *M.mus* liver. **F)** The fraction of CTCF-only, CTCF-YY1 and YY1-only binding events overlapping annotated repeat elements across species. Error bars represent the standard error of the mean among four species (*p <0.05, Wilcoxon rank-sum test, C vs. C+Y).


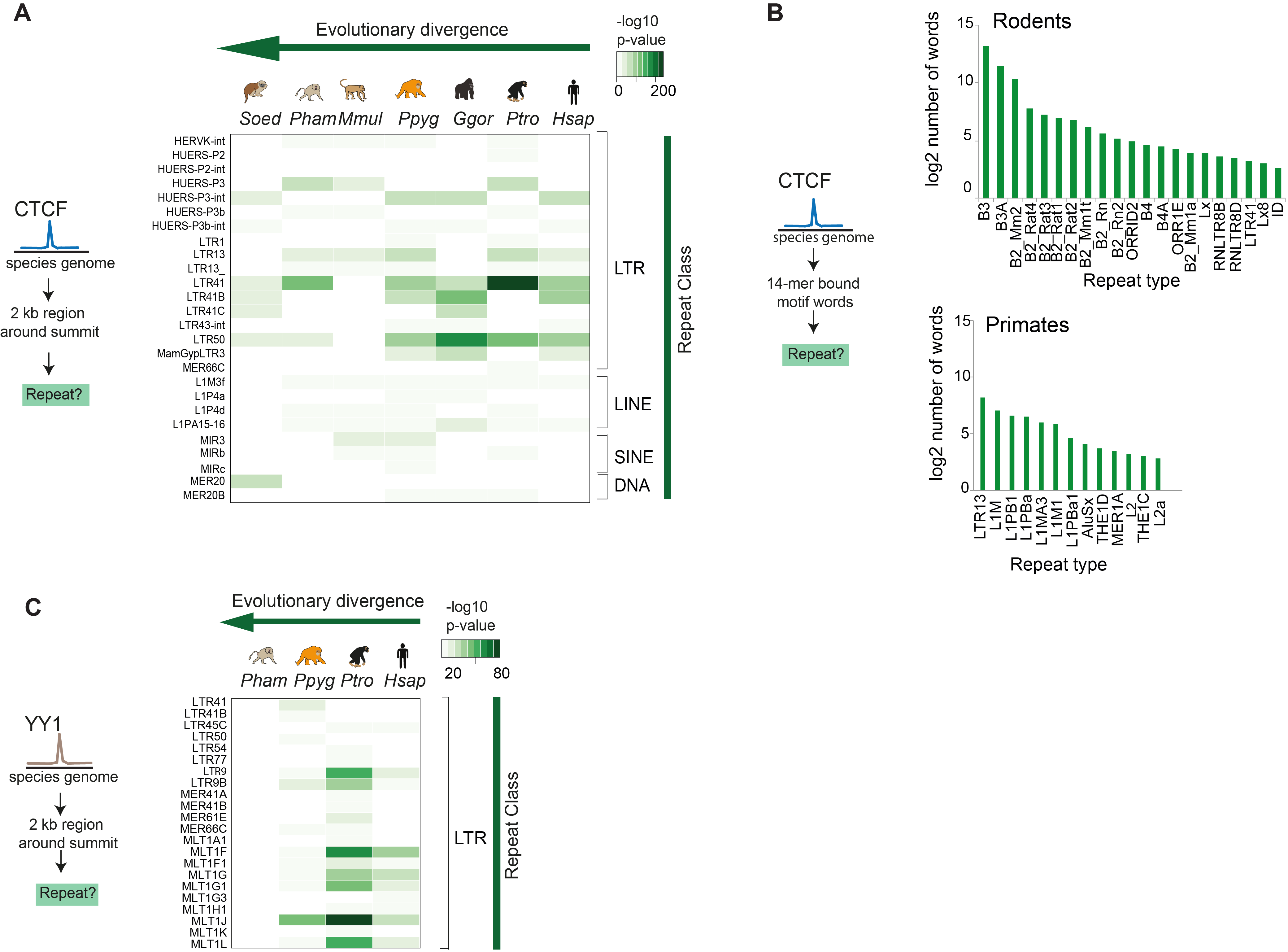


**Figure S4: CTCF and YY1 are associated with distinct repeat elements.** **A)** Repeat elements associated with CTCF binding based on their co-localisation with CTCF peak summits (p < 0.01). Repeats significant in at least one species are shown; the colour intensity represents the association’s significance. Repeat elements are sorted by repeat class and primate species by evolutionary distance from human. **B)** Log2 number of CTCF-bound motif words embedded within repeat elements in the rodent and primate lineages. **C)** Repeat elements associated with YY1 binding based on their colocalisation with YY1 peak summits (p < 0.01). Repeats significant in at least one species are shown; the colour intensity represents the association’s significance. Repeat elements are sorted by repeat class and primate species by evolutionary distance from human.


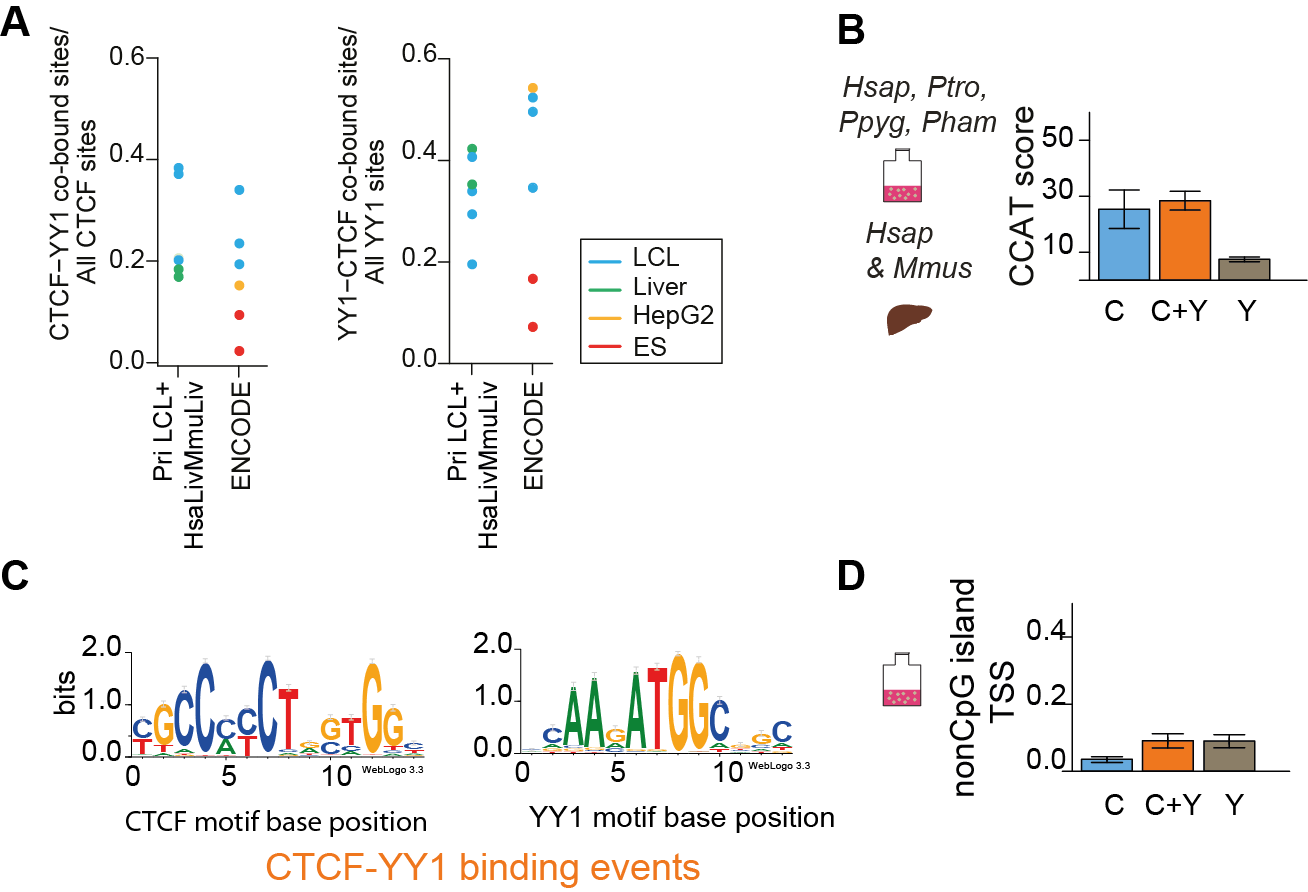


**Figure S5: CTCF and YY1 co-localise in various cell types at regions containing both CTCF and YY1 motifs. A)** Fraction of CTCF-YY1 binding events relative to all CTCF-bound regions; fraction of YY1-CTCF binding events relative to all YY1- bound regions in our primate LCL and mouse and human liver data as well as published data (Methods). LCL data from human and primate LCLs (blue), mouse and human liver (green), HepG2 (orange) and human and mouse embryonic stem cells (red). **B)** ChIP-seq enrichment in CTCF-only (C), CTCF-YY1 (C+Y) and YY1-only (Y) bound regions across primate LCLs (*H.sap*, *P.tro*, *P.pyg* and *P.ham*) and human and mouse liver as represented by the CCAT peak caller score. **C)** Motifs identified in CTCF-YY1 sites in primate LCLs and human and mouse liver. **D)** Fraction of binding events at non-CpG island transcription start sites in primate LCLs. Error bars represent the standard error of the mean among four species.

**
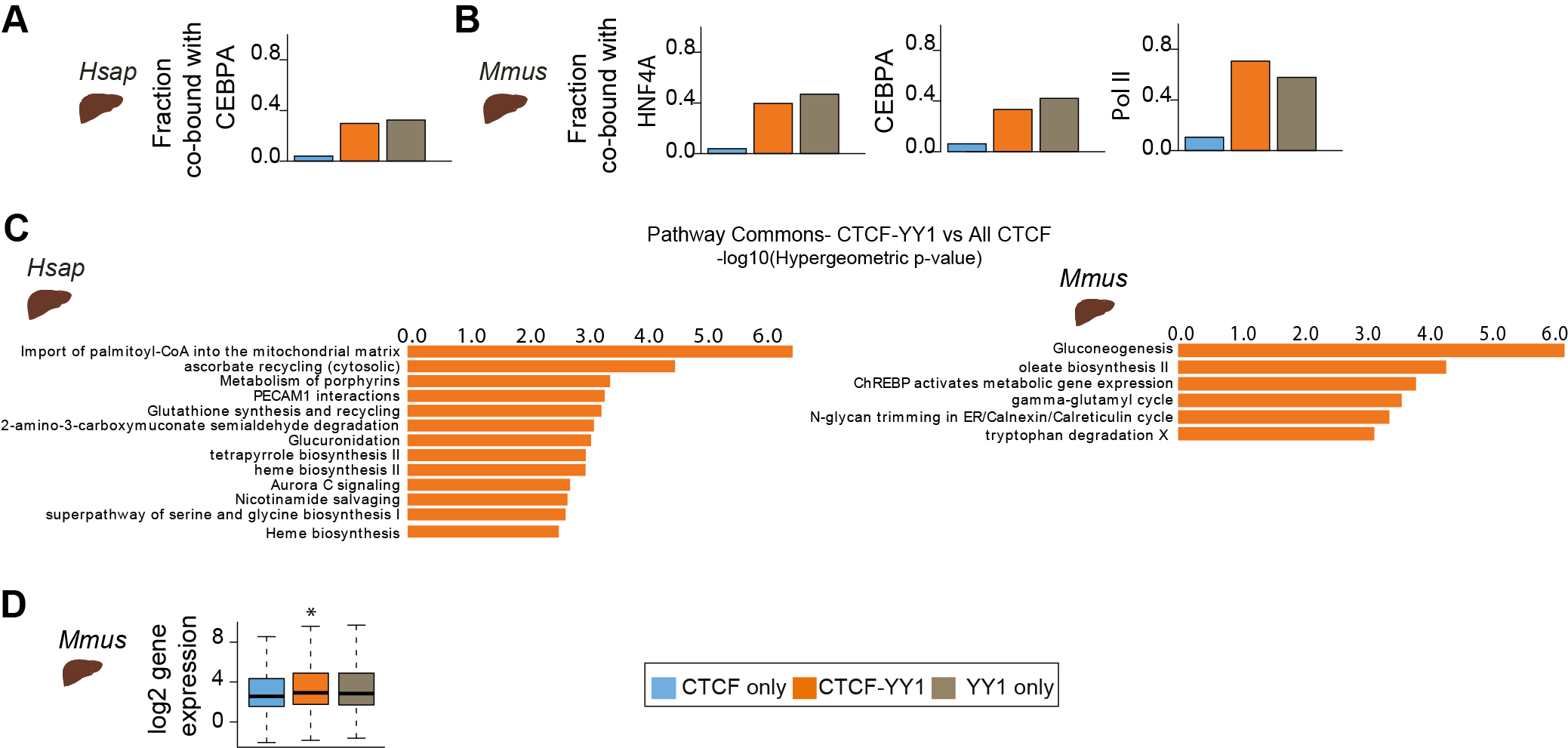
**

**Figure S6: CTCF-YY1 bound regions are associated with marks of active transcription. A)** Fraction of binding events associated with CEBPA in *H.sap* liver. **B)** Fraction of binding events associated with HNF4A, CEBPA and Pol II in *M.mus* liver. **C)** GREAT pathway analysis of CTCF-YY1 bound regions compared to all CTCF-bound regions in *H.sap* and *M.mus* liver. **D)** Expression level of genes overlapping CTCF-only, CTCF-YY1 and YY1-only binding events in *M.mus* liver. RNA-seq data from three independent individuals. (*p<0.05, Wilcoxon rank-sum test).

SUPPLEMENTAL TABLES

**Table S1: Primate cell lines and sources.** Individual cell lines used together with their respective sources. ECACC-European Collection of Cell Cultures, WTSI-Wellcome Trust Sanger Institute, NEPRC- New England Primate Research Center.

| **Species** | **Species** | **Cell line** | **Source** |
| --- | --- | --- | --- |
| Human 1 | Homo sapiens | 81.3 | ECACC |
| Human 2 | Homo sapiens | GM12878 | Coriell |
| Chimpanzee 1 | Pan troglodytes | EB176(JC) | ECACC |
| Chimpanzee 2 | Pan troglodytes | S0036222 | Coriell Institute (Yerkes Primates) |
| Chimpanzee 3 | Pan troglodytes | AG18359 | Coriell Institute, NIA Cell Repository |
| Gorilla 1 | Gorilla gorilla gorilla | EB(JC) | ECACC |
| Gorilla 2 | Gorilla gorilla gorilla | GG013 | Chris Tyler-Smith (WTSI) |
| Orangutan | Pongo pygmaeus pygmaeus | EB185(JC) | ECACC |
| Rhesus macaque 1 | Macaca mulatta | Mm173-2002 | Harvard Medical School, NEPRC |
| Rhesus macaque 2 | Macaca mulatta | Mm150-1999 | Harvard Medical School, NEPRC |
| Rhesus macaque 3 | Macaca mulatta | Mm71-2006 | Harvard Medical School, NEPRC |
| Rhesus macaque 4 | Macaca mulatta | Mm290-1996 | Harvard Medical School, NEPRC |
| Baboon | Papio hamadryas | 26 CB1 | ECACC |
| Cotton top tamarin | Sanguinus oedipus | B95-8 | ECACC |

**Table S2: ChIP-seq library summary.** CTCF and YY1 technical and biological replicate ChIP-seq libraries in each species. Numbers of sequencing reads (raw and post-processed) as well as the merged number of peaks for each dataset are shown**.**

**Table S3: CTCF and YY1 ChIP-seq peaks are associated with repeats. A)** Repeats significantly associated with CTCF ChIP-seq peaks (-log p >10) in at least one species are shown. **B)** Association of YY1 ChIP-seq data as for (A).

A) CTCF

| **Repeat Name** | **Species** | | | | | | | **Repeat Age** |
| --- | --- | --- | --- | --- | --- | --- | --- | --- |
|  | **Hsap** | **Ptro** | **Ggor** | **Ppyg** | **Mmul** | **Pham** | **Soed** | **(years)** |
| LTR13 | 42.17 | 55.40 | NA | 54.85 | 47.29 | 49.37 | NA | 29.55 |
| MER66C | NA | 20.51 | NA | NA | NA | NA | NA | 71.82 |
| HUERS-P3 | NA | 77.32 | NA | NA | 45.86 | 61.52 | NA | 78.18 |
| HUERS-P3-int | 65.31 | NA | 67.49 | 71.56 | NA | NA | 44.17 | 78.64 |
| L1M3f | 4.25 | 14.74 | 4.38 | 9.74 | 0.60 | 3.01 | NA | 85.00 |
| L1PA15-16 | 16.50 | 20.28 | 43.89 | 23.89 | 10.66 | 6.67 | NA | 85.00 |
| MER20 | NA | NA | NA | NA | NA | NA | 60.93 | 91.36 |
| LTR41 | 104.42 | 240.55 | 76.93 | 103.89 | NA | 123.45 | 33.53 | 109.55 |
| LTR50 | 84.21 | 113.58 | 162.17 | 93.46 | NA | 49.95 | 26.82 | 110.00 |
| LTR41B | 80.63 | NA | 111.06 | 72.81 | NA | NA | 31.56 | 112.73 |
| LTR41C | NA | NA | 77.48 | NA | NA | NA | 29.42 | 112.27 |
| MER20B | NA | 13.93 | 20.51 | 19.81 | NA | NA | NA | 119.55 |
| MIR3 | NA | NA | NA | 31.08 | 32.10 | NA | NA | 133.64 |
| MIRb | NA | 14.90 | NA | 9.82 | 15.48 | NA | NA | 134.09 |
| MamGypLTR3 | 33.91 | NA | 72.38 | 50.09 | NA | NA | NA | 140.45 |

B) YY1

| **Repeat Name** | **Species** | | | | **Repeat Age** |
| --- | --- | --- | --- | --- | --- |
|  | **Hsap** | **Ptro** | **Ppyg** | **Pham** | **(years)** |
| LTR9B | 9.16 | 45.89 | 15.71 | NA | 58.18 |
| MER61E | NA | 22.44 | NA | NA | 63.64 |
| LTR9 | 13.03 | 59.51 | 2.33 | NA | 84.09 |
| MLT1F1 | 7.81 | 16.49 | 4.63 | NA | 102.27 |
| MLT1F | 39.03 | 72.94 | 11.03 | NA | 105.91 |
| LTR41 | NA | NA | 13.45 | NA | 109.55 |
| MLT1G | 28.24 | 37.51 | 6.56 | NA | 118.18 |
| MLT1G1 | 16.97 | 49.78 | 2.20 | NA | 119.55 |
| MLT1J | 29.99 | 101.04 | 47.39 | NA | 127.73 |
| MLT1L | 22.38 | 64.00 | 12.82 | NA | 128.18 |
